# A Palaeogene stem crotaphytid (*Aciprion formosum*) and the phylogenetic affinities of early fossil pleurodontan iguanians

**DOI:** 10.1098/rsos.221139

**Published:** 2024-01-10

**Authors:** Simon G. Scarpetta

**Affiliations:** ^1^ Museum of Vertebrate Zoology, University of California Berkeley, 3101 UC Berkeley Road, Berkeley, CA 94720, USA; ^2^ Department of Environmental Science, University of San Francisco, San Francisco, CA 94117, USA

**Keywords:** squamata, phylogenetics, palaeontology, Palaeogene, Cretaceous

## Abstract

Pleurodonta is an ancient, diverse clade of iguanian lizard distributed primarily in the Western Hemisphere. Although the clade is a frequent subject of systematic research, phylogenetic resolution among the major pleurodontan clades is elusive. That uncertainty has complicated the interpretations of many fossil pleurodontans. I describe a fossil skull of a pleurodontan lizard from the Palaeogene of Wyoming that was previously allocated to the puzzling taxon *Aciprion formosum*, and provide an updated morphological matrix for iguanian lizards. Phylogenetic analyses using Bayesian inference demonstrate that the fossil skull is the oldest and first definitive stem member of Crotaphytidae (collared and leopard lizards), establishing the presence of that clade in North America during the Palaeogene. I also discuss new or revised hypotheses for the relationships of several early pleurodontans. In particular, I examine potential evidence for crown-Pleurodonta in the Cretaceous of Mongolia (*Polrussia*), stem Pleurodonta in the Cretaceous of North America (*Magnuviator*) and a stem anole in the Eocene of North America (*Afairiguana*). I suggest that the placement of the fossil crotaphytid is stable to the uncertain phylogeny of Pleurodonta, but recognize the dynamic nature of fossil diagnosis and the potential for updated systematic hypotheses for the other fossils analysed here.

## 1. Introduction

The palaeontological record provides information about life through time that cannot be acquired from study of the extant biota alone, and the utility of those data is predicated on accurate fossil identification and systematic diagnosis. Phylogenetic methods can eliminate biases that affect the accuracy of other diagnostics, such as modern biogeography or overall morphological resemblance [1], and allow fossils to be explicitly incorporated into analyses of divergence times [2] and biogeography [3]. However, there are many factors that influence systematic identifications of fossils that use phylogenetic methods. First, researchers can use either a phylogenetic analysis or an apomorphy-based diagnosis to place a fossil. When comparing phylogenetic analyses, the inferred tree topology and corresponding phylogenetic placement of fossil lineages can vary because of differences in the selection of individual characters for inclusion, data type (morphology-only or combined evidence), character-state scoring accuracy, scoring medium (computer tomography dataset, physical specimen, image), taxon sampling, analytical method (parsimony, maximum likelihood, Bayesian inference) and time calibration (uncalibrated, tip-dating, fossilized birth–death tip-dating). Thus, while phylogenetic methods provide a statistical approach to the systematic placement of fossils, that endeavour is dynamic rather than static, subject to continuous evaluation with different, updated, or new methods and matrices. This is particularly relevant for clades containing many systematically difficult fossils, as is the case for some lizard taxa, including pleurodontan iguanians.

Pleurodonta (Squamata: Iguania) is a diverse lizard clade containing approximately 1200 living species [4] that are distributed in North, Central and South America in all but the coldest environments and poleward latitudes. There are also tantalizing occurrences of pleurodontan lizards on the Fijian islands and Madagascar [5–8]. Pleurodonta includes well-known taxa such as anoles and horned lizards, and the clade has been frequently studied across biological disciplines, including phylogenetic [9], biogeographic [8,10], ecomorphological [11], palaeontological [12,13] and comparative [14] research. Although pleurodontan lizards have been a focal point for phylogenetic studies, the clade has perplexed systematists for decades; relationships among the family-level crown clades have been recalcitrant to all types of data and analysis that have been applied [8,9,13,15–26].

Phylogenomic timetrees indicate a Mesozoic origin for crown-Pleurodonta, with rapid divergences among most of the family-level crown clades during the Late Cretaceous (approx. 100–70 Ma) [8,15,27]. Those divergence times are not reflected by published fossils, and discrepancies between divergence-time analyses and first known fossil appearances are not unusual or unexpected. Discrepancies may result from a lack of known fossils near the age of a given node, adequate age control for known fossils and fossil misidentifications. Additionally, palaeontologists may not recognize fossils of crown clades as such due to taphonomic effects, an insufficient understanding of character evolution and variation, or phylogenetic uncertainty [2,28,29]. Although several of those issues are identifiable in Pleurodonta, particularly the persistently uncertain phylogenetic relationships among the family-level clades, the magnitude and ubiquity of the gap between Cretaceous divergence time estimates and known first fossil appearances is noteworthy, given the broad distribution and exceptional diversity of the extant species. For a few family-level clades, the first known fossils are from the early Eocene, approximately 56–48 Ma (e.g. Corytophanidae, [13,30,31]; Polychrotidae and Iguanidae, [32]). Surprisingly, for several clades (e.g. Phrynosomatidae, [12]; Liolaemidae, [33]; Tropiduridae, [34]) no fossils are known until the Neogene (23–5 Ma), others do not have a known pre-Pleistocene record (Opluridae, [35]), and some groups lack definitive fossils altogether (Hoplocercidae). Additionally, there are Late Cretaceous localities in North America that are within the extant range of crown pleurodontans (i.e. Phrynosomatidae and Crotaphytidae) that have produced putative stem pleurodontans or stem iguanians instead of crown pleurodontans [10,36].

**Figure 1.**
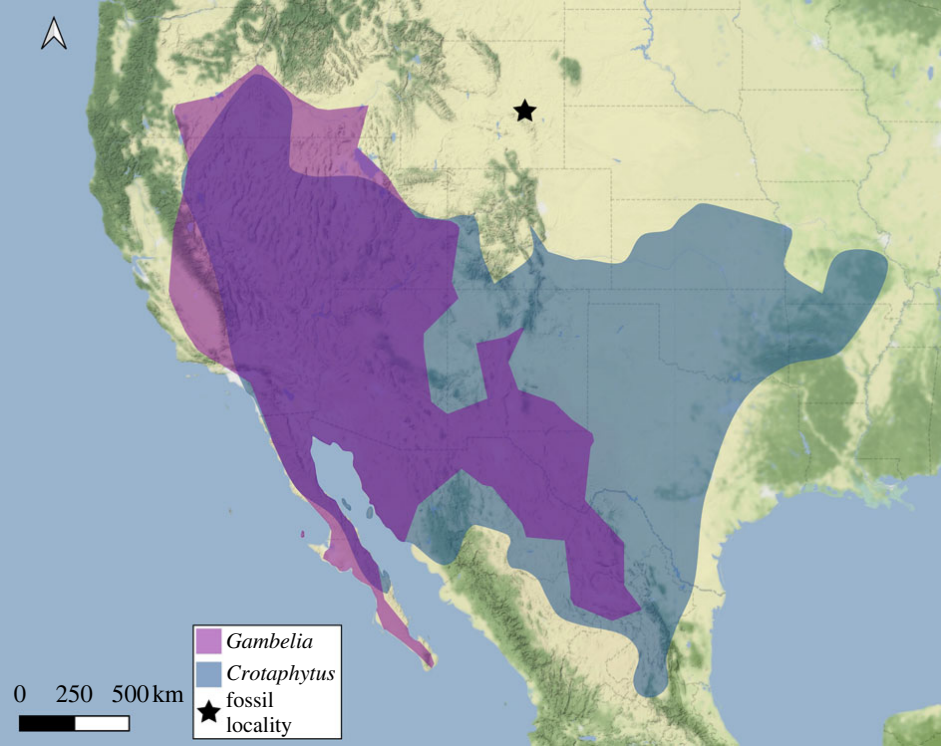
Extant range map for Crotaphytidae and locality of AMNH FR 11400. Range data were taken from GBIF.org [37] and manually filtered to remove outlier points (i.e. points outside of the known range of Crotaphytidae, for example, in South America or Europe). Point data were transformed to polygonal ranges using the concave hull algorithm in QGIS with α set to 0.15 for *Crotaphytus* and 0.2 for *Gambelia*. The black star labels the collection locality of *Aciprion formosum* AMNH FR 11400.

Perhaps the most curious example of a pleurodontan clade depauperate in pre-Neogene fossils is Crotaphytidae (collared and leopard lizards). Extant crotaphytid lizards are stocky, highly carnivorous and generally desert-dwelling lizards that inhabit much of the western and central continental USA and northern Mexico (figure 1) [38,39]. Total clade Crotaphytidae was estimated via divergence time analysis to be approximately 95 or 75 Myr old [8,15,27]. There are many known Pleistocene and Pliocene fossil occurrences of Crotaphytidae (see [40] for a summary of some of these), but few older occurrences. The oldest known crotaphytids are fragmentary dentary and maxilla fossils from the Miocene of Wyoming *ca* 17 Ma [12] and the early Pliocene of Nevada *ca* 4.7 Ma [41]. There is one older fossil from the Oligocene (*Crotaphytus oligocenicus*; [42]) originally reported to be part of Crotaphytidae that requires further study [38,40]. The bulbous tooth morphology of crown crotaphytids, while intraspecifically variable, has been considered diagnostic [41], and so early fossil crotaphytids should be identifiable based on their teeth if the evolution of the diagnostic morphology precedes the origin of the crown clade. An approximately 50–70 Myr gap between the putative age of total clade Crotaphytidae and the oldest known fossils is remarkable—many Palaeogene sedimentary deposits in the western and central USA are both fossiliferous and well-sampled and so diagnostic fossil crotaphytids could reasonably be expected from those deposits, but none are currently known.

**Figure 2.**
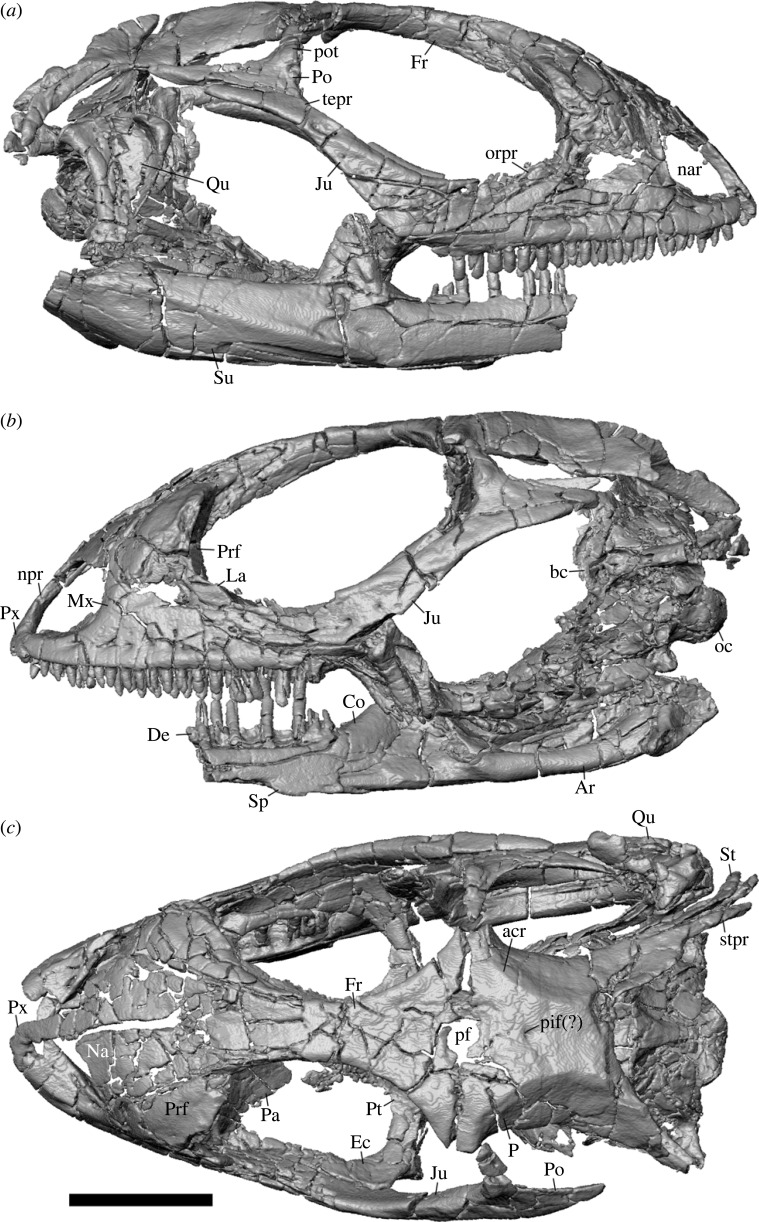
Skull of *Aciprion formosum* AMNH FR 11400. (*a*) Lateral right view; (*b*) left lateral view; (*c*) dorsal view. All images are surface renderings of segmented bones in orthographic view. Anatomical abbreviations: acr, adductor crest; Ar, articular; bc, braincase; Co, coronoid; De, dentary; Ec, ectopterygoid; Fr, frontal; Ju, jugal; La, lacrimal; Mx, Maxilla; nar, naris; Na, nasal; npr, nasal process of the premaxilla; oc, occipital condyle; orpr, orbital process; P, parietal; Pa, palatine; pf, parietal foramen; pif(?), pineal foramen; Po, postorbital, pot, postorbital tubercle; Prf, prefrontal; Pt, pterygoid; Px, premaxilla; Qu, quadrate; Sp, splenial; St, supratemporal; stpr, supratemporal process of the parietal; Su, surangular; tepr, temporal process. Scale bar = 5 mm.

**Figure 3.**
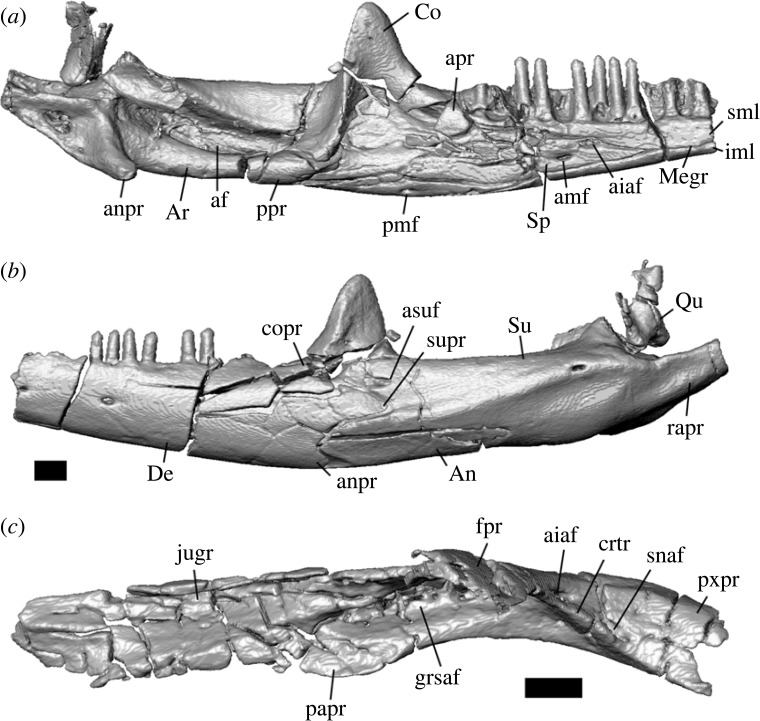
Left mandible of *Aciprion formosum* AMNH FR 11400 in (*a*) medial and (*b*) lateral view and (*c*) left maxilla in dorsal view. All images are surface renderings of segmented bones in orthographic view. Anatomical abbreviations: af, adductor fossa; aiaf, anterior inferior alveolar foramen; amf, anterior mylohyoid foramen; An, angular; apr, anterior process (of the coronoid); anpr, angular process; Ar, articular; asuf, anterior surangular foramen; Co, coronoid; copr, coronoid process (posterodorsal process of the dentary); crtr, crista transversalis; De, dentary; fpr, facial frocess; grsaf, groove containing the superior alveolar foramen; iml, inframeckelian lip; jugr, juga groove; Megr, Meckelian groove; papr, palatine process; pmf, posterior mylohyoid foramen; ppr, posterior process of the coronoid; pxpr, premaxillary process; Qu, quadrate; rapr, retroarticular process; sml, suprameckelian lip; snaf, subnarial arterial foramen; Sp, splenial; Su, surangular; supr, surangular process. Scale bars = 1 mm.

**Figure 4.**
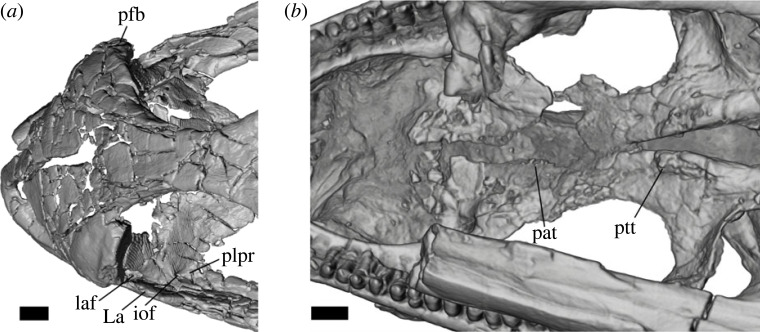
*Aciprion formosum* AMNH FR 11400 (*a*) snout in dorsal view; (*b*) palate in ventral view. Panel (*a*) is a surface rendering of segmented bones and (*b*) is a volume rendering of the scan; both are in orthographic view. Anatomical abbreviations: iof, infraorbital foramen; pat, palatine teeth; pfb, prefrontal boss; plpr, posterolateral process of the palatine; ptt, pterygoid teeth. Scale bars = 1 mm.

**Figure 5.**
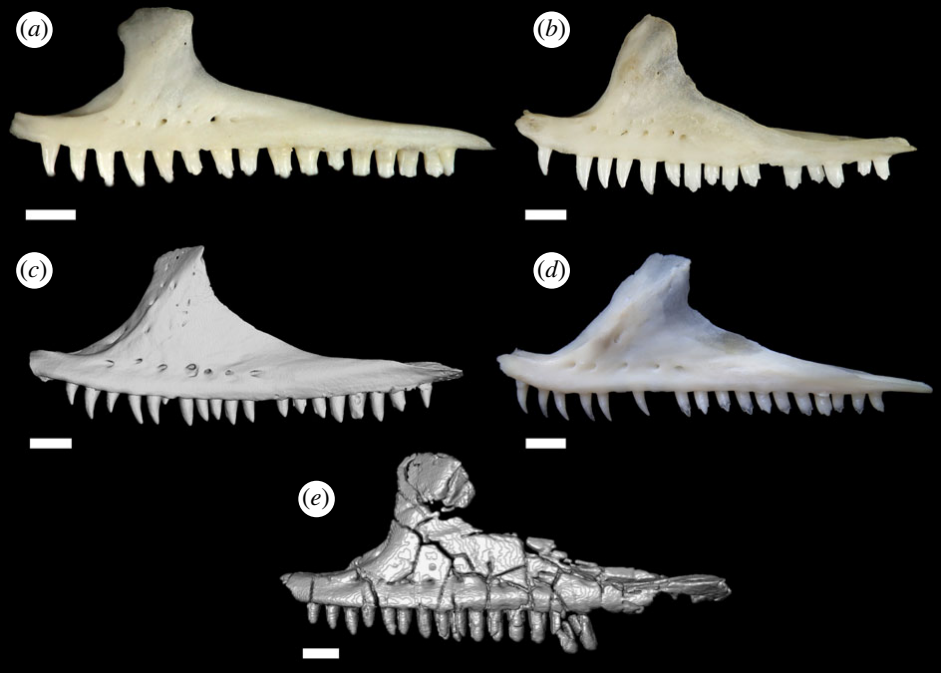
Comparison of maxillae and tooth morphology between extant crotaphytids and *Aciprion formosum* AMNH FR 11400. (*a*) *Crotaphytus collaris* TxVP M-9255; (*b*) *Crotaphytus bicinctores* TxVP M-8612; (*c*) *Gambelia sila* TNHC 95621 (from computed tomography scan); (*d*) *Gambelia wislizenii* TxVP M-8394; (*e*) *Aciprion formosum* AMNH FR 11400. Scale bars = 1 mm.

**Figure 6.**
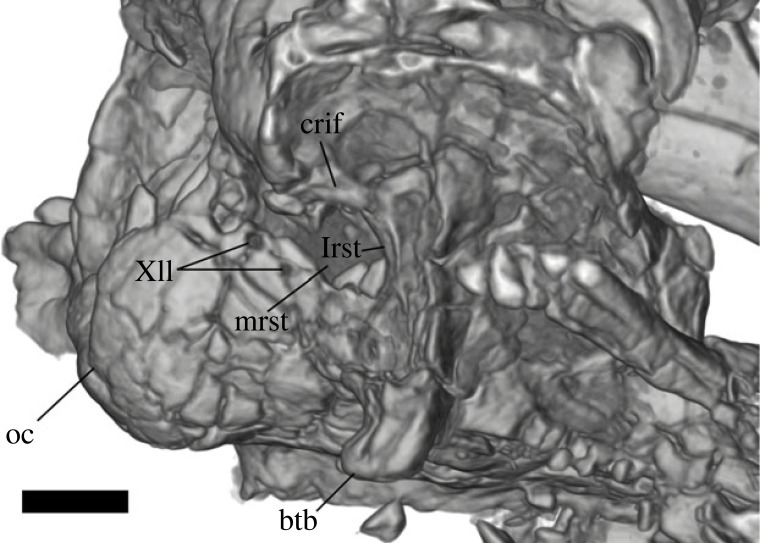
*Aciprion formosum* AMNH FR 11400 braincase in right ventrolateral view. The image is a volume rendering in orthographic view. btb, basipterygoid tubercle; crif, crista interfenestralis; lrst, lateral aperture for the recessus scalae tympani; mrst, medial aperture for the recessus scalae tympani; oc, occipital condyle, XII, hypoglossal foramina. Scale bar = 1 mm.

Here, I describe a largely complete and articulated skull (cranium and partial left and right mandibles; figures 2–6) of a stem crotaphytid lizard from the Palaeogene of North America. The fossil was previously assigned to the historically puzzling taxon *Aciprion formosum*. *Aciprion formosum* [43] was described based on a single partial left dentary, AMNH FR (American Museum of Natural History Fossil Reptiles) 1609 [43]. The fossil described here (AMNH FR 11400) has not been formally described, although it is probably the most complete known specimen referred to *Aciprion formosum* [21]. *Aciprion formosum* was included in several phylogenetic analyses of squamate reptiles [10,16,21,36,44] and in analyses of iguanian or pleurodontan relationships [8,22,23,45]. Several of those studies (e.g. [21] and any subsequent study that used that matrix) included the specimen described here.

Fittingly, there has been practically as much disagreement about the phylogenetic position of *Aciprion formosum* as there has been about the intrarelationships of Pleurodonta itself. *Aciprion formosum* has been placed in a polytomy at the crown-Pleurodonta node [21,22,36,45], as sister to pleurodontans excluding Crotaphytidae, Corytophanidae, Opluridae, Anolidae (see [46] for discussion of this name) and Polychrotidae [22], as sister to pleurodontans excluding Crotaphytidae, Corytophanidae, Iguanidae and Hoplocercidae [22], as sister to *Phrynosoma platyrhinos* [21], as a stem hoplocercid [8,10,23,44], as a stem member of the clade ((Polychrotidae, Corytophanidae), Hoplocercidae) [36], as a stem crotaphytid [22], or as a stem member of the clade ((Crotaphytidae, Leiocephalidae), Corytophanidae) [23]. *Aciprion formosum* was also previously considered to be a ‘messelosaurine', a hypothesized clade of extinct iguanians mostly composed of fossil pleurodontans from Europe that was reported to be closely related to Corytophanidae (basilisk lizards and relatives) (Rossman [47,48]). Several other proposed messelosaurines (i.e. species of *Geiseltaliellus*) were later placed in total clade Corytophanidae in phylogenetic analyses [13]. Many of the above phylogenetic studies used matrices (i.e. the matrix first published in [16], and that of [21]) that were primarily constructed to assess relationships among the major squamate clades, as opposed to a matrix specifically for iguanian or pleurodontan lizards.

As a part of the effort to describe and place *Aciprion formosum* AMNH FR 11400, to facilitate future systematic diagnoses of fossil pleurodontans, and for future use in combined-evidence analyses, I present an expanded version of the phylogenetic matrix published by Smith [13]). That matrix was originally constructed to infer relationships among iguanian lizards; in its present construction, it is primarily intended to place fossil pleurodontans in a phylogenetic framework in combined-evidence or topologically constrained phylogenetic analyses. The revised matrix contains an increased sample of extant and extinct iguanian lizards, updated character scores for some previously scored taxa, and several revised character states and characters. Finally, I discuss the phylogenetic affinities of several Cretaceous and Eocene pleurodontans included in the revised matrix. Specifically, I discuss extinct taxa that are well known, that have been used as node calibrations, and/or for which phylogenetic uncertainty has hindered systematic and biogeographic interpretation.

## 2. Material and methods

### 2.1. Specimen collection, geologic and palaeoenvironmental setting, and temporal constraint

AMNH FR 11400 was collected by Morris Skinner and party in 1959 near the town of Douglas in Converse County, Wyoming. The fossil locality, ‘Reno Ranch south of the Tower', is in the Palaeogene White River Formation (termed the White River Group in several other states, of which the Chadron and Brule are constituent formations). The locality spans both the Brule and the Chadron members of the White River Formation and both the Chadronian and Orellan North American Land Mammal Ages (NALMAs). Fossil-bearing Palaeogene sediments in the Douglas area span approximately 230 m and are well known for containing a wealth of fossil mammals, including camelids, lagomorphs, rodents and members of extinct clades like Leptomerycidae (ruminants) and Hyaenodontidae (carnivorous mammals) [49]. The badland outcrops of the lower Chadron member are characterized by clayey mudstones, sandy mudstones and sandstones, and the upper Brule member contains sandy mudstones, siltstones and sandstones [49]. White River localities near Douglas may have lacked standing water based on the near absence of freshwater snail fossils along with an abundance of terrestrial snail fossils [49,50]. The sedimentology and the density of fossil land snails were suggested to indicate a semi-arid, warm and temperate palaeoenvironment [49].

There are several datable ashes in the Douglas area. These include an ash variably dubbed the ‘Purple White layer’, ‘Persistent White layer', ‘Glory hole ash' or ‘5 ash’, which is just beneath the Chadronian-Orellan boundary and occurs near the top of the Reno Ranch south of the tower locality [51]. Five ^40^Ar/^39^Ar dates within that ash provided an age of 33.9 ± 0.06 Ma. Magnetostratigraphic correlation established that the Reno Ranch south of the tower locality is within chron 13, *ca* 33.214–35.102 Ma. [49,52–54]. Thus, 35.102–33.214 Ma, an age range encompassing the latest Eocene through the earliest Oligocene, should be considered the age range of AMNH FR 11400.

AMNH FR 11400 was deposited just before, during, or just after the Eocene–Oligocene transition (about 34–33.6 Ma), which was a global cooling period that resulted from decreasing pCO_2_ values due to silicate weathering, increased ocean productivity and carbon burial, and/or the development of the Antarctic Circumpolar current and corresponding changes in ocean circulation [55,56]. The impact of cooling varied globally and regionally and between marine and terrestrial environments. The magnitude of cooling in the continental interior of the USA was approximately 1.5–2 times as large as that of global ocean cooling [56], a temperature decrease of about 7°C. Although the age of the fossil is not precisely constrained around the cooling event, it is noteworthy that it was deposited adjacent to a period of global climate change that was amplified regionally and terrestrially.

### 2.2. Institutional abbreviations

AMNH FR, American Museum of Natural History, Fossil Reptiles Division, New York City, New York; FMNH, Field Museum of Natural History, Chicago, Illinois; IGM, Mongolian Institute of Geology, Ulaan Bataar, Mongolia; HLMD-Me, Messel Collection, Hessisches Landesmuseum, Darmstadt, Germany; IVPP, Institute of Vertebrate Paleontology and Paleoanthropology, Chinese Academy of Sciences; KNM-RU, National Museums of Kenya, Nairobi; LACM, National History Museum of Los Angeles County, Los Angeles, California (formerly Los Angeles County Museum); MOR, Museum of the Rockies, Bozeman, Montana; PTRM, Pioneer Trails Regional Museum, Bowman, ND, USA; UCMP, University of California Museum of Paleontology, Berkeley, California; TxVP, Texas Vertebrate Paleontology Collections, The University of Texas at Austin, Austin, Texas; USNM Smithsonian National Museum of Natural History, Washington, DC; UWBM, University of Washington Burke Museum, Seattle, Washington; YPM, Yale Peabody Museum, New Haven, Connecticut.

### 2.3. X-ray computed tomography

AMNH FR 11400 was scanned in 2005 for the Squamate Tree of Life project, on a Varian Medical Systems (Bio-Imaging Research, Inc) ACTIS computed tomography (CT) scanner with a FeinFocus X-ray source at the University of Texas at Austin High-Resolution X-ray Computed Tomography Facility. The cranium and mandible were scanned together with a voltage of 180 kV, amplitude of 0.133 mA, with no filter, no offset, air wedge, a source to object distance of 58 mm, and field of reconstruction 19 mm. The dataset contains 805 slices. The *X* and *Y* pixels are spaced at 0.018550 mm and the *Z* pixels are spaced at 0.039730 mm. S.G.S. performed the segmentation in Avizo Lite 2019.

### 2.4. Anatomical terminology and presentation of specimen

Anatomical terminology follows Evans [57] for most morphological features and Bhullar & Smith [58] for the terms infra- and supra-meckelian lip, which describe the ventral and dorsal flanges, respectively, that roof the Meckelian groove. AMNH FR 11400 is presented from the computed tomography data.

## 3. Phylogenetic analysis

### 3.1. Purpose of revised matrix in current and future studies, including addition of terminal taxa and characters

Expanding the matrix of Smith [13] has two main purposes. First, an updated matrix serves to place *Aciprion formosum* AMNH FR 11400 and other fossil iguanians (particularly pleurodontans) in the present study and in future studies. Second, the matrix will be used in the future for combined-evidence analyses (both calibrated and uncalibrated) using Bayesian inference—I did not attempt to create a new or heavily revised morphological matrix (for a recent character matrix, see [22]) or make inferences about iguanian relationships based on unconstrained analyses, nor did I perform parsimony analyses. Regardless, the number of extinct taxa included in the present study is nearly or more than double that of most published studies that included extinct iguanians (e.g. [17,21,45]) and comparable to a recent study [22] that focused more on fossil acrodontans.

### 3.2. Sampling strategy of terminal taxa

I scored an increased sample of extant pleurodontan and acrodontan lizards, as well as many extinct iguanians, for the matrix created by Smith [13]. The original matrix included 39 total taxa, including 29 extant iguanians and four extinct iguanians. The revised matrix includes 133 total taxa, including 102 extant iguanians and 27 extinct iguanians, with an emphasis on fossil pleurodontans. There are at least three species for each extant pleurodontan family in the dataset. I included extinct taxa known from articulated fossil skulls or skeletons, or multiple isolated fossils that were explicitly associated with each other based on a combination of size, frequency, morphology and locality; see Smith [31] for a discussion of specimen association). For the extant species, I sampled broadly within each clade but did not attempt comprehensive coverage, particularly for speciose clades like Agamidae or Anolidae that contain hundreds of species. All extant specimens were scored from physical skeletons or skeletons visualized from CT scans. For many extinct taxa, I scored specimens from the physical fossil or CT scans that I visualized, but several taxa were scored from published illustrations, descriptions, or video visualizations of processed CT datasets when the original dataset could not be acquired (see electronic supplementary material, appendix S1 for specifics for all specimens). Extinct taxa scored from images and descriptions include *Afairiguana avius*, *Anchaurosaurus gilmorei*, *Anolbanolis banalis* and *Anolbanolis geminus*, *Oreithyia oaklandi*, *Sauropithecoides charisticus*, *Suzanniwana revenata* and *Queironius praelapsus*. For a few fossils (e.g. *Pumilia novaceki* and *Gambelia corona*), CT data will be acquired in the future to augment the character scores presented here.

Most included fossils are stem members of an extant pleurodontan family, stem pleurodontans or stem acrodontans. Several included extinct taxa are crown members of an extant pleurodontan family, but are articulated skull or skeleton fossils that are referable to relatively less speciose groups like Crotaphytidae (e.g. AMNH FR 11400, *Gambelia corona*), Corytophanidae (e.g. *Geiseltaliellus*) and Iguanidae (e.g. *Armandisaurus*, *Pumilia*). Relatively complete fossils from very speciose groups, like the many known amber anoles [11], were excluded. The use of an anole-specific matrix yielded highly uncertain results for amber anoles [11,59], so the matrix of Smith [13] as it was previously and presently constructed is not likely to be an appropriate dataset for inferring the phylogenetic position of those fossils with respect to the extant taxa. I did not include isolated and fragmentary fossils, e.g. the fossil *Uma* from Scarpetta [60], the fossil phrynosomatine from Scarpetta [12], the fossil *Pristidactylus* from Albino *et al*. [61], fossil *Liolaemus* from Albino [62], and the acrodontans *Jeddaherdan* from Apesteguía *et al*. [63] and *Gueragama* from Simões *et al*. [64]. Analysis of the last two taxa is more appropriate for a matrix with more Acrodonta-specific characters (e.g. [22,65]).

Note that this revised matrix is not intended for inferring interspecific relationships of extant iguanian lizards. Creating a phylogenetic matrix for a diverse group, like Iguania, with character constructions simultaneously informative of relationships among all species and between all family- or subfamily-level clades would be impractical, if not impossible [66]. This is especially true with respect to exceptionally diverse clades like Liolaemidae, Anolidae and Agamidae. I emphasize that the increased sampling here is intended to capture variation within each of the major clades to systematically and reliably place fossils included in the matrix.

### 3.3. Character sampling

For most analyses, I excluded all non-osteological characters (67–80, 82, 130–152) (*exclude* command in MrBayes), because those characters could not be scored for nearly all of the fossils, and the primary intent of the matrix is to place the fossils in with this matrix in the context of combined-evidence or topologically constrained phylogenetic analyses. I also performed topologically unconstrained analyses that included all of the characters (see electronic supplementary material, figures). Several of the non-osteological characters were previously scored on specimens of the stem corytophanid *Geiseltaliellus maarius* [13]. Preliminary analyses in which non-osteological characters were included did not change the phylogenetic position of *Geiseltaliellus maarius*. I also excluded character 61 (quadrate orientation) because I was unable to consistently score the character for iguanians as presently or originally [67] constructed, and I did not reformulate the character. I revised several characters in Smith [13] and added three characters (new characters 153, 154 and 155) based on recently published literature [68] and revision of one character (for character revisions and new characters, see appendix A). Thus, there were 119 variable characters in the dataset that did not contain non-osteological characters.

### 3.4. Topology

I used two tree hypotheses produced from target sequence capture datasets containing ultraconserved elements (UCEs) [9] or loci collected via anchored hybrid enrichment (AHEs) [15] as topological scaffolds, similar to the methodology of Scarpetta [69]. I constrained most relationships among subfamily-level agamid clades and among the family-level pleurodontan clades, but allowed intra-group relationships to be estimated in the analyses. I also constrained Brookesiinae and Chamaeleoninae in the scaffold analyses, but relationships among chameleons were otherwise unconstrained given uncertainty about relationships (compare [70] and [27]). I also performed unconstrained analyses. Given the uncertain relationships among the family-level clades of Pleurodonta across analyses and datasets, I stress that the phylogenetic position of some of the fossils included here (e.g. *Polrussia* IGM 3/73) may fluctuate in the context of other tree hypotheses or analysis types, although I propose that many fossils consistently recovered within the same clades (e.g. *Aciprion formosum* AMNH FR 11400, *Armandisaurus explorator* AMNH FR 8800) are phylogenetically stable (see Results).

### 3.5. Uncalibrated Bayesian analyses

Uncalibrated analyses were performed in MrBayes v. 3.2.7 [71]. The analyses were conducted for two runs of 4 000 000 generations, with four chains, and sampling every 1000 generations. The symmetric Dirichlet hyperprior was set at infinity and an Mk model of character evolution was used. Trees were summarized as 50% majority-rule consensus trees. Results were visualized in Tracer v. 1.7.1 [72] to confirm sufficient effective sample size values greater than 200, which were used to infer Markov chain Monte Carlo (MCMC) convergence. Analyses were performed on the CIPRES supercomputer cluster [73]. The 50% majority-rule consensus trees are in electronic supplementary material, file S2.

### 3.6. Fossilized birth–death analyses

I performed relaxed clock analyses using a fossilized birth–death (FBD) model to explore the incorporation of that model and stratigraphy on topology estimation for the extinct taxa [74]. The effect of the FBD model on fossil placement, rather than divergence time estimation, was the purpose of these analyses, so I do not discuss the associated divergence times (see electronic supplementary material, figures S3–S4, S7, S13 for node ages). FBD analyses were performed in MrBayes v. 3.2.7 for two runs, each of 25 000 000 generations, with five chains, sampling every 1000 generations. The symmetric Dirichlet hyperprior was set at infinity. The posterior distributions of trees were summarized as 50% majority-rule consensus trees. I used default priors for the FBD processes: a speciation prior with an exponential distribution with a mean of 10, and *β* distributions for the extinction and fossilization priors with *α* = *β* = 1. The sample probability was set to 0.051 (the proportion of sampled extant iguanian species; 102/2003) and the sample strategy was set to ‘fossiltip', which assumes that each fossil is a terminal tip and not a direct ancestor of an extant terminal. I used the independent gamma rates (IGR) clock prior and an IGR variance prior with an exponential distribution with a mean of 10 (the default). The clock rate prior was set to 0.00183 (set using a lognormal distribution with a mean of −6.30, the natural log of the clock rate). The clock rate was established by performing a strict clock analysis of the data for 2 000 000 generations, with a tree height set to an exponential distribution with a mean of 1, and dividing the resulting tree height (0.353) by the mean age of the root calibration (193.2 Ma) (methodology of [75]). For the strict clock analysis, I used the scaffold from Streicher *et al*. [9]. For the FBD analyses, all age distributions for extinct taxa were set with uniform priors of the minimum and maximum age of the fossil(s) scored for each taxon (see appendix B for age range information for each extinct taxon). Results are presented as 50% majority-rule consensus trees. The 50% majority-rule consensus trees are in electronic supplementary material, file S2.

## 4. Results

### 4.1. Systematic palaeontology

Squamata Oppel 1811

Iguania Cuvier 1817

Pleurodonta Cope 1864 (=Iguanidae *sensu* Schulte *et al*. [24] and Iguanoidea *sensu* Daza *et al*. [17])

Crotaphytidae Smith and Brodie [76] *sensu lato*

Referred specimen: AMNH FR 11400

Figures 2–6

### 4.2. Diagnosis

AMNH FR 11400 is diagnosed as a crotaphytid lizard based on the following combination of character states: pleurodont tooth implantation (figure 3*a*; a morphological state consisting of two separate state transformations according to [68], and that was considered an apomorphy of Lepidosauria in [77]); presence of a splenial (figure 3*a*; the absence of splenial is an apomorphy of Rhynchocephalia and some crown squamates such as many amphisbaenians, and the presence of a splenial is a plesiomorphy of Squamata; [68,78]); mobile frontoparietal joint and embryonic fusion of parietals (figure 2*c*) and separation of pterygoids from vomers by palatines (figure 4*b*), all of which are apomorphies of Squamata [79]; parietal foramen at frontoparietal suture and presence of prefrontal boss (figures 2*c* and 4*a*, respectively; apomorphies of Iguania, [21]; the former is exclusive to Iguania and the latter is also present in Teiidae); presence of separate foramina for the subnarial artery and anterior inferior alveolar nerve on the dorsal surface of the premaxillary process of the maxilla (figure 3*c*; apomorphy of Pleurodonta; [13]); the presence of palatine teeth (figure 3*b*; apomorphy of Crotaphytidae, also present in and a potential apomorphy of the clade (Leiosauridae, Opluridae); [13,38]); a closed but unfused Meckelian groove (figure 3*a*; present in Crotaphytidae, Corytophanidae, Phrynosomatidae, Liolaemidae, *Anolbanolis* and *Caeruleodentatus*, among crown pleurodontans; [12,13,31]); a deep groove for the superior alveolar foramen on the dorsal surface of the maxilla (figure 3*c*; present in Crotaphytidae, Corytophanidae and Leiocephalidae; [12,31]), a posteriorly deflected temporal ramus of the jugal (figure 2*a,b*; present in Crotaphytidae, absent in Corytophanidae; [31]), a trapezoidal parietal table (figure 2*c*; present in Crotaphytidae, absent in Corytophanidae; [31]), and a jugal that is broadly exposed above the orbital process of the maxilla (figure 2*a*,*b*; present in Crotaphytidae, absent in Corytophanidae; [13,31]). AMNH FR 11400 and Crotaphytidae also share recurved mesial teeth and at least some recurved distal teeth (figures 2*a*,*b*, 3*a* and 5), though these were not used as phylogenetic characters. AMNH FR 11400 differs from crown-Crotaphytidae in lacking a discrete posteroventral (quadratojugal) process of the jugal, a postorbital that broadly underlaps the corner of the frontoparietal suture and a large palatine process of the maxilla [13,38].

AMNH FR 11400 differs from the holotype specimen of *Aciprion formosum,* AMNH FR 1609, with respect to tooth morphology. The teeth of the holotype are proportionally thicker and closer-spaced than those of AMNH FR 11400 and the secondary cusps are better developed. The Meckelian groove is closed but unfused in both specimens and the suprameckelian lip is well-developed dorsal to the closure.

### 4.3. Remarks

Tooth morphology is insufficient evidence to establish a new taxon for AMNH FR 11400, especially given an exclusive relationship with Crotaphytidae. Extant crotaphytids are well known for possessing intra- and inter-specifically variable tooth morphology (figure 5; [12,38,41,80,81]). Thus, the referral to *Aciprion formosum* is provisionally retained. If new material attributable to *Aciprion formosum* is discovered from the type locality (the Oligocene White River Formation in Logan County, Colorado) that indicates that AMNH FR 1609 and AMNH FR 11400 belong to separate taxa, then a new taxon should be erected for AMNH FR 11400. Compared with extant crotaphytids, the relatively parallel-sided, regularly spaced teeth of AMNH FR 11400, particularly the dentary teeth, are most similar to *Gambelia wislizenii* (figure 5), although not all specimens of that taxon have relatively gracile teeth and the teeth of *Gambelia* are often more recurved [12]. The teeth of AMNH FR 11400 lack the general irregularity and bulbous tooth bases that are characteristic of *Crotaphytus* and some specimens of *Gambelia sila*.

### 4.4. Description

Almost all cranial elements are fully or partially preserved, and most are in articulation and in the natural anatomical location (figure 2). Many bones are fractured into smaller pieces. There is a thin sheet of bone just anterior to the palatines that is probably a piece of the septomaxilla, but I have not identified the piece definitively because it is incomplete and located dorsal and posterior to the expected location of the septomaxilla. No portions of the stapes, squamosal, epipterygoid, vomer or hyoid were preserved. Additionally, while it is very likely given the morphology of the postorbital that there was no separate postfrontal, I cannot confirm the absence of a small, separate postfrontal element.

Although the exact ontogeny of AMNH FR 11400 is not clear, the morphology of the fossil indicates an individual that is neither neonatal or juvenile—the specimen is well into skeletal ontogeny (i.e. is skeletally mature). Morphologies that support skeletal maturity include the absence of a frontoparietal fontanelle, a roughly square parietal that is not exceptionally wide, fusion of the basioccipital and otoccipital (especially near the foramen magnum) near fusion of the sphenoid and basioccipital, and fusion between the supraocciptal and left prootic (on the right side, the suture between those elements appears to be more clearly visible) [82–84]. Fusion between the otoccipital and prootic is not clear because of bone breakage.

### 4.5. Premaxilla

Most of the nasal process and the left side of the main body of the premaxilla are preserved (figure 2*c*). Although the right ventral portion of the process is missing, it is evident that the nasal process is narrow and gradually tapers anteriorly to posteriorly. The nasal process is exposed dorsally over the nasals for the entire length of the process. Two partial teeth are present, but a total tooth or tooth position count is not possible.

### 4.6. Maxilla

Both maxillae are present and mostly complete (figure 2*a*,*b*). The facial process is complete on the left side only, and is narrow in the anterior–posterior dimension. There are two foramina on the dorsal surface of the premaxillary process, one for the subnarial artery and the other for the anterior inferior alveolar nerve. There is a deep groove on the dorsal surface of the alveolar plate in which the superior alveolar foramen sits. The orbital process is narrow and uniform in width, and has a deep jugal groove on its surface that occupies much of the width of the process. There is no ridge to medially buttress the articulation between the jugal and the maxilla. The palatine process is symmetrical in shape but is small, barely extending medially beyond the alveolar plate. There are 21 tooth positions and 18 teeth on the right maxilla, and 22 tooth positions and 16 teeth on the left maxilla. Fragmentation of the maxillae in multiple pieces made it difficult to count lateral nutrient foramina, but there are at least five foramina just dorsal to the tooth row on the left maxilla, with at least two additional foramina located dorsal to that row. There are at least five foramina dorsal to the tooth row on the right maxilla as well.

### 4.7. Nasal

Both nasals are present and nearly or fully complete, but fragmented (figure 2*c*). Anteromedially, the nasal is located ventral to and articulates with the nasal process of the premaxilla, and anterolaterally, the nasal is in contact with the facial process of the maxilla. The short anterior process of the nasal contains the articular facet for the nasal process of the premaxilla on its dorsal surface. Posteriorly, the nasal articulates tightly with frontal and prefrontal. There is no frontonasal fontanelle.

### 4.8. Prefrontal

Both prefrontals are preserved (figure 2*c*). The right prefrontal is relatively more fragmented and the left prefrontal is missing most of the posterior process. The prefrontal boss is evident but does not extend far laterally or posteriorly from the body of the bone (figure 4*a*).

### 4.9. Lacrimal

Fragmentation of the lacrimal and the orbital process of the jugal made differentiating between the two bones difficult on the right side of the skull (figure 2*a*), but on the left side the lacrimal is more discrete (figure 2*b*). The lacrimal contacts the facial process of the maxilla anteriorly, the orbital process of the maxilla ventrally and the jugal posteriorly. Medially, the lacrimal bounds the lacrimal foramen, which is laterally bounded by the ventral process of the prefrontal. The lacrimal foramen is large but not substantially larger than the infraorbital foramen. The lacrimal is laterally exposed dorsal to the orbital process of the maxilla.

### 4.10. Jugal

Both jugals are preserved, but the orbital process of each is more fragmented than the rest of the bone (figure 2*a*,*b*). Still, the orbital process of the jugal clearly has a substantial lateral exposure dorsal to the orbital process of the maxilla. The postorbital (temporal) process is posteriorly deflected. The angle of the jugal is sharp, but there is no discrete quadratojugal process (jugal spur). There are at least three foramina on the lateral surface of the bone.

### 4.11. Postorbital

Both postorbitals appear to be nearly complete, if fragmented into several pieces (figure 2). The postorbital is triradiate with dorsal, posterior and anterior processes. The posterior process is broader and longer than the other two processes. There is a distinct tubercle around mid-height of the dorsal process. The dorsal process was separated from the frontal and parietal during fossilization, but evidently lacks articulation surfaces that would strongly underlap the frontoparietal suture.

### 4.12. Frontal

The frontal is nearly complete, but the anterior processes are broken and separated into many smaller pieces (figure 2*c*). The frontal is azygous and is constricted in the interorbital region relative to both the anterior and posterior portions of the bone. The supraorbital flanges are poorly developed. The dorsal surface of the element is mostly flat, but is slightly concave in the middle posterior of the bone, near the parietal. The parietal foramen invades the posterior face of the frontal.

### 4.13. Parietal

The parietal is complete except for the left postparietal (supratemporal) process, which is missing the posteroventral end (figure 2*c*). The right postparietal process is broken off and slightly separated from the parietal table. The parietal foramen is present and located at the boundary of the parietal and the frontal, invading the margins of both bones. A separate pineal foramen may be present. The parietal table has a trapezoidal shape. The adductor crests are poorly developed. The descensus parietalis has a broad lateral extent, and faces ventrolaterally.

### 4.14. Supratemporal

The right supratemporal is present and complete (figure 2*c*). The element articulates along most of the lateral surface of the postparietal process of the parietal. The bone is mediolaterally very thin, and is slightly taller in the middle of the bone relative to the anterior and posterior portions, which taper to blunt tips.

### 4.15. Palatine

The palatines are broken into pieces (figures 2*c* and 4). The anterior process is largely missing on each palatine, and the posterior process of the left palatine is incomplete. Still, it is possible to distinguish a deep choana in ventral view and, importantly, several palatine teeth on the ventral surface of the right palatine (figure 4*b*). The lateral and posterolateral processes are well-preserved and are both well-developed on the left palatine; on the right palatine, the former is present but the latter is broken. The lateral process encloses the infraorbital foramen anteriorly and dorsally, while the posterolateral process encloses the foramen posteriorly and to an extent ventrally (figure 4*a*). The infraorbital foramen is otherwise ventrally and laterally enclosed by the dorsal surface of the orbital process of the maxilla.

### 4.16. Pterygoid

Both pterygoids are preserved and largely complete. There are large patches of pterygoid tooth attachment sites on the ventral surface of both pterygoids, especially on the left element (figure 4*b*). One large pterygoid tooth is present on the left pterygoid. The contact between the palatine and the pterygoid is straight and anteromedially directed.

### 4.17. Ectopterygoid

The ectopterygoid is preserved on each side of the skull (figure 2*c*), and each one is broken into several large blocks. The bone is triradiate, with long anterior and medial processes and a substantially shorter lateral projection. The anterior process tapers in width anteriorly, and is set in a well-developed jugal groove on the dorsal surface of the maxilla; laterally the anterior process contacts the medial surface of the jugal. The medial process is bifurcate and is composed of two processes that clasp the pterygoid flange. The small lateral process is broken on the left ectopterygoid, but on the right side does not attain a marked lateral exposure between the jugal and the orbital process of the maxilla. There is a large foramen on the dorsal surface of the ectopterygoid medial and anterior to the lateral process.

### 4.18. Braincase

Almost all individual elements of the braincase are preserved in part except for the left prootic, which is missing most of the lateral face (figure 2). The sphenoid is largely complete, but like the rest of the braincase, is broken into many smaller pieces. Still, major anterior openings are evident within the pituitary fossa, including the anterior vidian canal, the abducent foramen (cranial nerve VI) and the internal carotid foramen. Although fragmented, the posterior processes of the sphenoid extend posteriorly to contribute to the basal tubercle (figure 6). The right basal tubercle is large and well-preserved, if slightly detached from the rest of the braincase. The basipterygoid processes are fragmentary and somewhat removed from the rest of the bone. The cephalic condyle is mostly complete, although the ventral basioccipital contribution is fragmented. Otherwise, the separate components of the cephalic condyle appear largely fused together. The supraoccipital is complete and broken into only a few pieces. The anterior surface of the supraoccipital is flared dorsally, nearly reaching the parietal. There is no supraoccipital crest distinct from the rest of the anterior surface. The osseous labyrinth is hardly elevated above the otooccipital.

The prootic crest is long, extending from the paroccipital process to the sphenoid, although that ventralmost extent is poorly preserved, particularly on the right side of the braincase. There are no alar processes of the prootic and no evident supratrigeminal process to bisect the incisura prootica. On the otooccipital, the crista interfenestralis is present and separates the fenestra ovalis from the recessus scali tympani (figure 6). The fenestra ovalis is relatively large, and is about equal in size to the medial aperture of the recessus scali tympani. The lateral aperture of the right otooccipital is less distinct because the crista tuberalis is largely missing and the ventral portion of the crista interfenestralis is broken. The vagus foramen (cranial nerve X) is present on the left otooccipital but does not appear to be preserved on the right otoccipital (figure 6). There are at least two hypoglossal foramina (cranial nerve XII) on the right otooccipital but that portion of the bone is missing on the left otooccipital. The paroccipital process is complete on the right side, and while the left process is comparatively less fragmented, it is missing its posterior portion. The right paroccipital process is long and articulates with the right supratemporal and postparietal (supratemporal) process of the parietal.

### 4.19. Quadrate

The right quadrate is complete (figure 2*a*), but only the mandibular condyle of the left quadrate is present (and is attached to the preserved portion of the left mandible, figure 3*a*,*b*). The quadrate possesses well-developed medial and lateral concha. The column is slightly curved posteriorly.

### 4.20. Mandible

Both dentaries are present, but more of the left dentary is present both anteriorly and posteriorly (figures 2*a*,*b* and 3*a*,*b*). On the left dentary, the Meckelian groove is closed but not fused by the infra- and supra-meckelian lips at the 10th most distal tooth position and anterior to that position. The surangular and angular processes are well-developed, but the surangular process is larger (figure 3*b*). The surangular process extends posteriorly just past the apex of the coronoid process of the coronoid, while the angular process extends to the apex. The right dentary has a ventrally and posteriorly well-developed intramandibular lamella that mediates the articulation between the splenial and the anteromedial process of the coronoid. The left dentary, anteromedial process of the coronoid, and posterior portion of the splenial are all fragmented such that determining the geometry of their articulation was difficult. There are two preserved nutrient foramina on the lateral surface of the dentary.

The splenial is present on both mandibles but is incomplete on the right mandible (figure 2*b*), and the left splenial is fragmented into several pieces (figure 3*a*). On the left mandible, the splenial extends posteriorly to the anterior margin of the coronoid process of the coronoid, and anteriorly to the ninth most distal tooth position. The anterior inferior alveolar foramen is fully enclosed by the splenial. The posterior mylohyoid foramen is present and located ventral and slightly posterior to the anterior inferior alveolar foramen (figure 3*a*).

There is no anterolateral process to articulate with the lateral surface of the dentary, and correspondingly the dentary lacks a lateral facet for the coronoid (figure 3*b*). The articulation between the anterior process of the coronoid and the splenial mostly occurs internally, such that the anteromedial process of the coronoid is visible for only one or two of the distalmost tooth positions.

The angular extends anteriorly to the mesial margin of the penultimate tooth position, and posteriorly to the adductor fossa. The posterior mylohyoid foramen is located anterior to the apex of the coronoid process of the coronoid. The anterior surangular process is located dorsally between the coronoid process of the coronoid and the surangular process of the dentary. There is a large, anteromedially extending angular process (figure 3*a*).

### 4.21. Dentition

Mesial maxillary teeth are unicuspid with crowns that taper to a point (figures 2*a*,*b* and 5*e*). Both the tooth shaft and tooth crown are recurved for many teeth, especially mesially, and recurvature is more pronounced on the mesial teeth. On the maxillae (only the distal dentary teeth are preserved) unicuspid crowns transition quickly to tricuspid crowns around the eighth tooth position. All preserved dentary teeth are tricuspid (figures 2*a*,*b* and 3*a*,*b*). Most teeth are weakly tricuspid, potentially because of preservation, but the accessory crowns of some maxillary teeth are more pronounced. The mesialmost preserved teeth of the left dentary are slightly recurved. Distal dentary and maxillary teeth are slightly wider mesiodistally than the mesial teeth, but no teeth have bulbous tooth bases compared with the rest of the tooth shaft or the crown.

### 4.22. Non-clock phylogenetic analysis

#### 4.22.1. Unconstrained

Inter-family relationships of Pleurodonta were generally similar to those found by Smith [13]. However, the analysis did not encounter issues with the monophyly of Pleurodonta (electronic supplementary material, figures S1 and S2), a result encountered in the Bayesian analyses of Gauthier *et al*. [21] and Smith [13]. Many nodal posterior probabilities (pp) were low (electronic supplementary material, figures S1 and S2).

#### 4.22.2. Constrained

**Figure 7.**
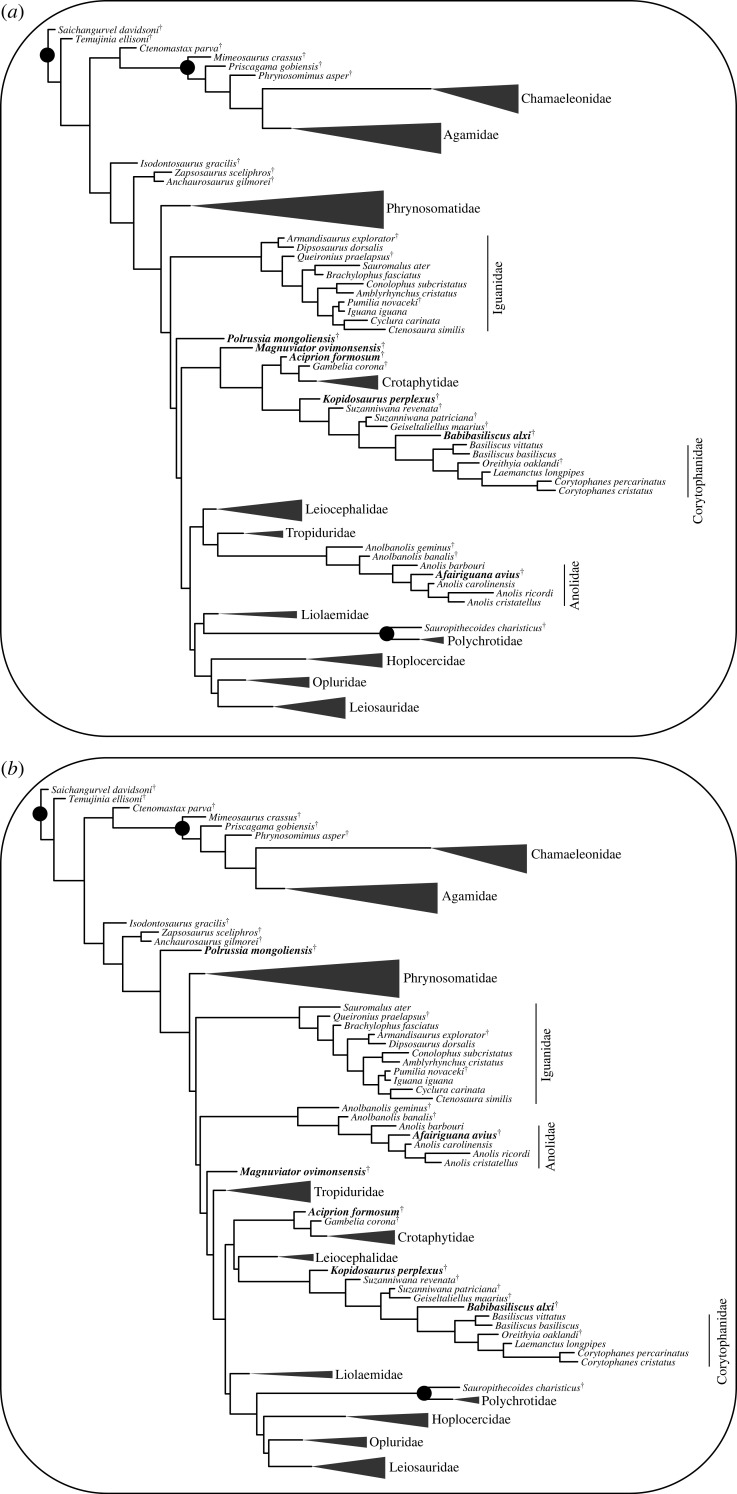
Uncalibrated phylogenies emphasizing the position of extinct taxa with the constraints from (*a*) Streicher *et al*. [9] and (*b*) Burbrink *et al*. [15]. Extinct taxa in bold are those that are specifically reviewed in the Discussion, and a ^†^ labels an extinct taxon. Family-level clade names label crown clades, and are collapsed when no extinct taxa was inferred to be part of the crown clade. Black circles label relationships between an extinct taxon and its sister taxon supported with greater than 0.95 posterior probability.

Relationships of many of the extinct taxa were similar to those hypothesized in earlier phylogenetic analyses or apomorphy-based diagnoses (figure 7). For example, *Isodontosaurus* and *Zapsosaurus* were placed as stem pleurodontans, as in some recent phylogenies (e.g. [21]). *Armandisaurus* was placed as the sister taxon of *Dipsosaurus*, as in all published phylogenies that included that extinct species [21,44]. *Sauropithecoides* was hypothesized to be a stem polychrotid by Smith [32] and *Pumilia* was suggested to be the sister taxon of *Iguana* by Norell [81], both using qualitative apomorphic diagnoses. Both of those hypotheses were corroborated by the analyses here. Similarly, *Oreithyia* was hypothesized to be a crown corytophanid by Smith [32] and here was placed in crown-Corytophanidae in all uncalibrated analyses. Several taxa (*Mimeosaurus*, *Phrynosomimus* and *Priscagma*) were previously placed as stem acrodontans and generally in the clade Prisagamidae; here, those taxa were again estimated to be stem acrodontans but were instead placed in a single grade or a grade and a clade containing only two of the species on the stem of Acrodonta. *Aciprion formosum* was consistently placed as the sister taxon of extant Crotaphytidae.

### 4.23. Fossilized birth–death analyses

#### 4.23.1. Unconstrained

The tree topology was again similar to Smith [13], although Phrynosomatidae was paraphyletic in this analysis—*Phrynosoma* was outside of Phrynosomatidae. The other main difference was that in this analysis, all ‘isodontosaurids' and *Magnuviator* were inferred to be crown pleurodontans, and were placed as a grade of successive sister taxa of the clade including Phrynosomatidae, Liolaemidae, Opluridae and Tropiduridae (electronic supplementary material, figure S3). In the unconstrained analysis with all characters, isodontosaurids were placed as stem pleurodontans (electronic supplementary material, figure S4).

#### 4.23.2. Constrained

**Figure 8.**
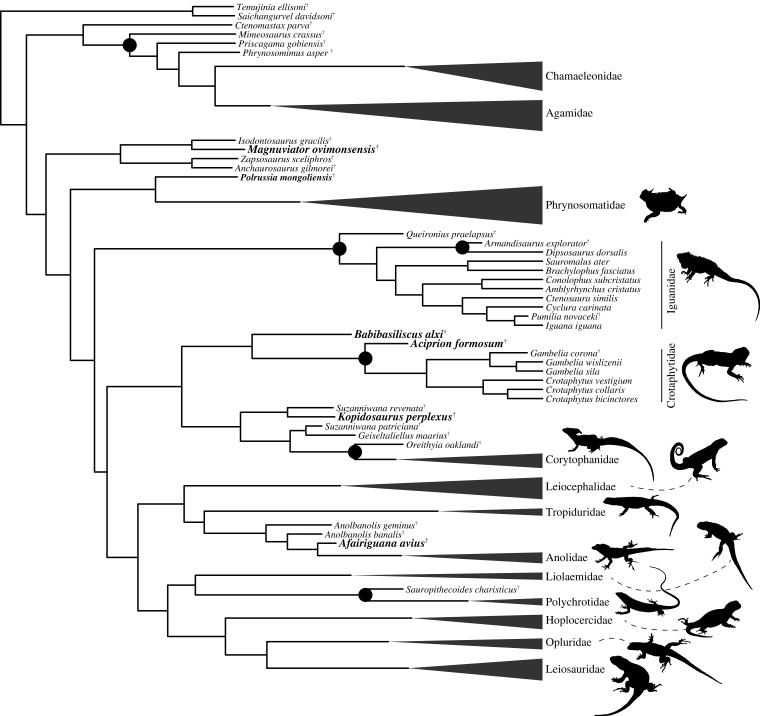
FBD phylogeny with the constraints from Streicher *et al*. [9]. The positions of the extinct taxa in the FBD tree with the constraint from Burbrink *et al*. [15] are nearly identical (see Results). Family-level clade names label crown clades, and are collapsed when no extinct taxa were inferred to be part of the crown clade. Black circles label relationships between an extinct taxon and its sister taxon supported with greater than 0.95 posterior probability; the same nodes occur in the FBD analysis with the constraints from Burbrink *et al.* [15]. A ^†^ labels an extinct taxon. All illustrations were created by the author using CC0, CC-BY or CC-BY-NC, or images by the author or Drew R. Davis. Attributions are in the electronic supplemental material, appendix S2.

The placement of many fossils was the same in the uncalibrated (figure 7) and FBD analyses (figure 8), but the placement of a few extinct taxa differed. In the constrained FBD analyses *Magnuviator* was placed on the stem of Pleurodonta instead of in the crown, similar to the topologically constrained analyses from DeMar *et al*. [10]. Similarly, in the constrained FBD analyses *Oreithyia* was placed on the stem instead of in the crown of Corytophanidae and *Queironius praelapsus* was inferred to be on the stem rather than in the crown of Iguanidae. *Oreithyia* was hypothesized to be a crown corytophanid and *Queironius* a crown iguanid by Smith [32]. *Babibasiliscus* was inferred to be a stem crotaphytid or on the stem of (Corytophanidae, Leiocephalidae) in the FBD analyses with the constraints from Streicher *et al*. [9] (figure 8) and Burbrink *et al*. [15] (electronic supplementary material, figure S4), respectively. *Babibasiliscus* was a stem corytophanid in all of the unconstrained analyses and was placed as sister to *Laemanctus* by Conrad [45]. *Gambelia corona* was placed as sister to crown-Crotaphytidae in the uncalibrated analyses but was the sister taxon of extant *Gambelia* in the FBD analyses. *Afairiguana avius* was placed in crown-Anolidae in the non-FBD analyses but as a stem member of the clade in the FBD analyses. Trees with associated divergence times (electronic supplementary material, figures S7 and S13) and nodal posterior probabilities (electronic supplementary material, figures S8–S12, S14).

## 5. Discussion

### 5.1. Why has *Aciprion formosum* been so difficult?

*Aciprion formosum* AMNH FR 11400 is a relatively complete skull that is clearly a pleurodontan, and the fossil does not lack character data (see below) or have unusual character states that have hindered the systematic placement of some other Palaeogene fossil pleurodontans (e.g. *Cypressaurus*, *Parasauromalus*, *Kopidosaurus*; [32,69]). So why has determining the systematic position of *Aciprion formosum* and AMNH FR 11400 specifically been so difficult?

With respect to the taxon *Aciprion*, many Palaeogene fossils were referred to *Aciprion formosum* that are almost certainly not the same species or genus as the holotype. In one of the few diagnoses presented for *Aciprion*, including both fossils referred to *Aciprion formosum* and *Aciprion* sp., Estes [40] remarked on the generalized iguanid (= pleurodontan) cranial features of known fossils, and suggested that most observed character states were ancestral. That said, Estes [40] also suggested that *Aciprion* was closely related to the putatively early-diverging morunasaurines (= hoplocercids) based on the unfused closure of the Meckelian groove anterior to the splenial, a result obtained in many later analyses (e.g. [10,44]). Recent phylogenomic trees find Hoplocercidae to be nested deep in crown-Pleurodonta [9,15]. Contra Estes [40], most hoplocercids I have examined have a broadly open Meckelian groove. Given that and the relative paucity of character data on the holotype of *Aciprion* (*Aciprion formosum* AMNH 1609, a partial dentary), there is little evidence that any fossil ascribed to *Aciprion* should have been referred to the taxon.

Subsequently, very few authors have undertaken systematic revisions of fossils referred to *Aciprion*. One such effort by Smith [32] revised the taxonomy of several fossils from the Eocene of North Dakota that were tentatively assigned to cf. *Aciprion* sp. by Smith [85], and erected the genus *Oreithyia* to accommodate those and newly described fossils. The new taxon was hypothesized to be a corytophanid, a result which I obtained here. Otherwise, there do not appear to be any other systematic reassessments of the Oligocene and Eocene fossils referred to *Aciprion formosum* or *Acriprion* sp. besides the present study. Although several studies included specimens of *Aciprion formosum* in phylogenetic analyses (see references in Introduction), those studies did not formally diagnose the taxon or any specimen referred to the taxon. In sum, determining the systematic relationships and/or creating a systematic diagnosis for a taxon to which fossils from several independent lineages have probably been referred is not possible, and so the relationships of *Aciprion* have been and will continue to be unresolvable until most fossils are systematically reassessed. The results of any phylogenetic analysis that included *Aciprion formosum* pertain only to the specimens scored for those studies; AMNH FR 11400 here and in studies that used the matrix from Gauthier *et al*. [21], and AMNH FR 8717 in Conrad [16] and studies that used that or modified versions of that matrix. Finally, I reiterate that it is not clear that AMNH FR 11400 is the same species or genus as the holotype, although I retain the assignment for the time being because of known inter- and intra-specific variation in tooth morphology in crown crotaphytids [12,38].

The authors of one study suggested that missing data could account for difficulties in placing *Aciprion formosum* (AMNH FR 11400 was the specimen scored), and that the taxon was ‘primitive and would therefore root deep in the tree' [21, p. 24]. In that study, *Aciprion formosum* AMNH FR 11400 was placed in a polytomy at the base of Pleurodonta or as sister to *Phrynosoma platyrhinos*. AMNH FR 11400 is listed on DigiMorph.org as a corytophanid, possibly based on the suggestions of Rossman [47,48]. In Gauthier *et al*. [21], 273 of 610 total characters (45%) were coded for *Aciprion formosum* AMNH FR 11400, and in the present study, 79 of 119 total, variable characters (66%) were coded for the specimen. Rather than missing data, I suggest that the matrix from Gauthier *et al*. [21], which was primarily designed for assessing higher-level relationships among squamates, did not contain enough characters that were informative for specific intrarelationships of crown-Pleurodonta (see [69]). Prior analyses that used that matrix probably encountered the same issue (e.g. [23,44]).

The other factor that has hindered interpretation of fossils referred to *Aciprion* is the uncertain phylogenetic relationships among the family-level clades of Pleurodonta, an issue that has complicated systematic placements of many Late Cretaceous and Palaeogene pleurodontans [16,21,69,86]. Interpretation of a morphological feature as apomorphic or plesiomorphic is contingent on tree topology, and so precise systematic allocation of a fossil is difficult when tree topology is uncertain. That situation is exemplified by the analyses of Scarpetta [23] (using the matrix from Gauthier *et al*. [21]). In that study, *Aciprion formosum* AMNH FR 11400 was placed as a stem hoplocercid or as an outgroup to (Corytophanidae, (Leiocephalidae, Crotaphytidae)) in combined-evidence analyses using two different filtering strategies of the same UCE dataset, which produced slightly different maximum-likelihood topologies [23]. The use here of an Iguania-specific dataset with relatively dense sampling compared with other matrices and iterations of the same matrix appears to have alleviated the issue.

### 5.2. Evolution of tooth morphology in Crotaphytidae

*Aciprion formosum* AMNH FR 11400 is identifiable as a crotaphytid based on a suite of morphological characters that are only known in that clade, but the specimen lacks the bulbous or mesiodistally expanded teeth that are characteristic of extant *Crotaphytus* and some individuals of *Gambelia* [12,41]. The fossil, the first definitive stem member of Crotaphytidae, provides evidence that that morphology is an apomorphy of crown-Crotaphytidae. That hypothesis could be further tested by the discovery of additional fossil crotaphytids and the systematic reassessment of other known fossils, such as other fossils that were allocated to *Aciprion formosum*. The discovery of fossils of stem-*Crotaphytus* and stem-*Gambelia* would be especially useful to elucidate the evolution of tooth morphology in crotaphytids. I suggest that for the time being mesiodistally expanded teeth should not be used in isolation to identify a fossil to crown-Crotaphytidae in an apomorphy-based diagnosis.

AMNH FR 11400 has some recurved teeth in the middle of the tooth row, which is characteristic of Crotaphytidae, particularly *Gambelia*, but the fossil does not have recurved distal teeth. The recurvature is consistent with known crotaphytids but does not add any new data on the teeth of crotaphytids. The fossil possesses tricuspid teeth, like most pleurodontans and all *Crotaphytus* and *Gambelia*. Tooth cusps are often but not always more pronounced in *Gambelia* than in *Crotaphyus* [12,38]; the teeth of AMNH FR 11400 are weakly to moderately tricuspid as in some individuals of both modern genera.

### 5.3. Biogeography of *Aciprion formosum* AMNH FR 11400 and total clade Crotaphytidae

AMNH FR 11400 is the oldest known crotaphytid and is also the first definitive stem crotaphytid (but see *Babibasiliscus* section below). There are no extant crotaphytids in Wyoming where the fossil was collected, but crotaphytids are known from Neogene localities in Wyoming [12], so AMNH FR 11400 does not represent a major range extension for the total clade. Douglas, Wyoming, is approximately 400 miles from the nearest modern occurrences of crotaphytids in Colorado or Idaho (figure 1; GoogleEarth), but the locality of AMNH FR 11400 is not further north or west than any record of an extant crotaphytid (figure 1). AMNH FR 11400 indicates that, minimally, the ancestral range of total clade Crotaphytidae included mid-latitude North America, an unsurprising result based on the modern biogeography of the clade. What continues to be surprising is the lack of fossil crotaphytids from earlier in the Cenozoic, given the putative Late Cretaceous age of total clade Crotaphytidae [15,27] and the prevalence of other fossil pleurodontans, like corytophanids and anolids, in middle latitudes of North America during the early Eocene [31]. Besides AMNH FR 11400, the geographical whereabouts of early crotaphytids are still unknown. Were crotaphytids excluded from known Eocene localities based on ecological factors, such as the megathermal habitats that existed at the time, or competition from other lizards? Have past occurrences not yet been detected, i.e. would more sampling of Palaeogene localities in the modern range of Crotaphytidae produce fossil crotaphytids? Or, have known fossils been misidentified (see *Babibasiliscus* section below)? Answers to these questions await the discovery of additional fossils and the results of future research efforts to systematically reassess known fossils. For now, *Aciprion formosum* AMNH FR 11400 provides the first conclusive evidence of total clade Crotaphytidae in North America during the Palaeogene.

### 5.4. Phylogenetic relationships and biogeography of some other early fossil pleurodontans

#### 5.4.1. *Polrussia mongoliensis* IGM 3/73: a crown pleurodontan in Central Asia?

*Polrussia mongoliensis* is a Late Cretaceous taxon known from the Ukhaa Tolgod and adjacent localities in the Gobi Desert of Mongolia that was first described by Borsuk-Bialynicka and Alifanov [87]. Many noteworthy fossil lizards have been collected from that area, including some other iguanians used here (e.g. *Isodontosaurus*, *Temujinia*), as well as scincomorph (e.g. *Slavoia*), anguimorph (e.g. *Gobiderma*) and gekkotan (e.g. *Norellius*) lizards. I note that Simões *et al*. [68] questioned whether IGM 3/73 is actually *Polrussia mongoliensis*, because the specimen has pterygoid teeth and the holotype lacks them. Although intraspecific variability in the presence of pterygoid teeth is known in at least some extant pleurodontans (e.g. some *Anolis*, [88]), the phylogenetic results from this study should for the time being be applied to IGM 3/73 only and not to the holotype. Only IGM 3/73 was used here because CT data was available for that specimen but not the holotype.

Another potential issue involves the ontogeny of specimens of *Polrussia*. The relatively squared shape of the parietals of *Igua minuta* and possibly both specimens *Polrussia mongoliensis* were hypothesized to indicate juvenile specimens [68]. The shape of the parietal in iguanians experiences marked ontogenetic shape changes, like in other squamates. However, the closure of the frontoparietal fontanelle that accompanies the ontogenetic shape shift is arrested in iguanians, and some otherwise skeletally mature specimens, especially phrynosomatids, may retain a relatively square parietal table and a large frontoparietal fontanelle [82,83,84]. The latter feature was previously suggested to be an apomorphy of Isodontosauridae, a hypothesized clade of extinct iguanians that included *Polrussia*, *Isodontosaurus* and *Zapsosaurus* [21]. The shape of the parietal of *Polrussia mongoliensis* IGM 3/73, a wide rectangle, is certainly reminiscent of juvenile individuals of modern iguanians. However, the full closure and fusion of the Meckelian groove across the dentary anterior to the splenial that is present in IGM 3/73 would be highly unusual in a juvenile individual. IGM 3/73 was treated as a mature specimen in the analyses here.

*Polrussia* was inferred to be a stem or crown pleurodontan in previous analyses. Gobiguanidae, another hypothesized clade of extinct iguanians that included *Polrussia*, was inferred to be sister to (Polychrotidae, Hoplocercidae) by Daza *et al*. [17]. *Igua* and *Polrussia* were within crown-Pleurodonta and placed as sister to *Chalarodon* in the analyses of Conrad *et al*. [89]. The holotype of *Polrussia* was placed as the sister taxon of Tropiduridae (represented by a single species) in the non-clock analyses of Simões *et al*. [68], but as sister to crown-Pleurodonta in the FBD analyses of that study. IGM 3/73 was placed in Isodontosauridae by Gauthier *et al*. [21]. Isodontosauridae was sister to crown-Pleurodonta or in a polytomy with many pleurodontans in the analyses of Gauthier *et al*. [21].

Here, *Polrussia mongoliensis* IGM 3/73 was inferred to be a crown pleurodontan sister either to Phrynosomatidae (both topologically constrained FBD analyses), to crown pleurodontans excluding Iguanidae and Phrynosomatidae (uncalibrated analyses with the [9] scaffold), nested in crown-Phrynosomatidae (uncalibrated analyses with the [15] scaffold), or sister to a clade containing Phrynosomatidae, Tropiduridae, Opluridae, Leiocephalidae and Liolaemidae (uncalibrated unconstrained analysis). All of these relationships were estimated with low posterior probability support values (less than 0.5 pp). There do not seem to be individual characters or character suites that clearly place IGM 3/73 with respect to the extant pleurodontan families, and no characters that clearly demonstrate that IGM 3/73 is *not* a member of an extant family. However, the presence of at least four characters in combination is potentially suggestive of a place in the crown clade, and could support an exclusive relationship with Phrynosomatidae over alternative hypotheses. IGM 3/73 has unicuspid teeth, a closed and fused Meckelian groove, a discrete and relatively high-angled dorsal lamina of the facial process of the maxilla, and it lacks a posterolateral process of the palatine. Among pleurodontans, unicuspid teeth in mature individuals are known in a few extant phrynosomatids (e.g. *Callisaurus draconoides*, *Sceloporus gadoviiae*, [12]) and leiosaurids. IGM 3/73 was scored as having unicuspid teeth here; if any future analysis determines that the specimen is a juvenile, then this character scoring should be reassessed.

A closed and fused Meckelian groove is present in species of Leiocephalidae, Anolidae, Iguanidae, Polychrotidae, Tropiduridae, Leiosauridae, Liolaemidae (variably present) and Corytophanidae (variably present), and is considered apomorphic of some of those clades or specific members of those clades [12,13]. Although infrequently recognized, phrynosomatid lizards of the genus *Urosaurus* (particularly *Urosaurus ornatus*) can also have a closed and fused Meckelian groove, indicating that this feature probably appeared early in the evolution of pleurodontans if Phrynosomatidae is sister to the rest of crown pleurodontans [9], or that the morphology is even more plastic than previously thought (see [18]). A dorsal lamina of the facial process of the maxilla is present in several pleurodontans. Members of Anolidae have a low-angled lamina of the facial process with respect to the horizontal plane of the maxilla, whereas a higher-angled lamina is present in Phrynosomatidae and Tropiduridae [13]. Among pleurodontans, some leiosaurids, tropidurids and most phrynosomatids lack a posterolateral process of the palatine [13]. Leaving aside the unicuspid teeth of IGM 3/73, the fused Meckelian groove, dorsal lamina of the facial process at a high angle, and the lack of a posterolateral process of the palatine are together consistent with either Tropiduridae (as found by [68] for the holotype of *Polrussia* using a different matrix) or Phrynosomatidae. A sister taxon relationship with Tropiduridae seems extremely unlikely, though not impossible, given that extant tropidurids are completely restricted to continental South America and some adjacent islands, and that no fossil tropidurids have been found outside South America.

The placement of *Polrussia mongoliensis* IGM 3/73 in crown-Pleurodonta here and in several other studies presents an intriguing departure from many recent biogeographic hypotheses for iguanian lizards. If the placement of IGM 3/73 as a stem phrynosomatid is correct, the basal divergence between Phrynosomatidae and other crown pleurodontans probably occurred in central Asia, and so the hypothesized ancient rapid radiation of Pleurodonta in the Western Hemisphere [9,26] was restricted to *non*-phrynosomatid pleurodontans instead of all crown pleurodontans. This hypothesis is also interesting given the absence of known fossil phrynosomatids in North America during the Palaeogene and Cretaceous, although that could be the result of sampling artefact or inability to identify known fossils associated with Pleurodonta, most of which are isolated and highly fragmentary [36]. Currently, the oldest known definitive phrynosomatids are from the Miocene of Florida [90] and Wyoming [12]. The presence of crown pleurodontans in Late Cretaceous deposits of central Asia was previously suggested by Alifanov [91], who described *Desertiguana gobiensis* from a partial mandible and interpreted the new taxon as a member of Phrynosomatidae. Several other Late Cretaceous iguanians (*Anchaurosaurus*, *Igua* and *Zapsosaurus*) were assigned to Phrynosomatidae in the same publication [91]. The only phylogenetic analysis to include *Desertiguana* [22] placed that taxon on the stem of Pleurodonta rather than in the crown, although this could be explained by the material being limited to a mandible. I did not include *Igua* here but both *Anchaurosaurus* and *Zapsosaurus* were placed as stem pleurodontans, as in most other studies.

#### 5.4.2. *Magnuviator ovimonsensis* and the early dispersal of total clade Pleurodonta to the Western Hemisphere

Specimens of *Magnuviator ovimonsensis* were described from the Late Cretaceous of Montana by DeMar *et al*. [10]. Until the discovery of *Magnuviator*, few, if any, fossil iguanians were known from the Late Cretaceous of North America [10], and certainly none as large and well-preserved as the exquisite skull and skeletons of *Magnuviator*. In the unconstrained analysis from DeMar *et al*. [10], *Magnuviator* was inferred to be the sister taxon of Temujiniidae, and *Magnuviator* + Temujiniidae was placed as sister to Pleurodonta or to all other iguanians. Analyses with molecular scaffolds placed *Magnuviator* as sister to crown-Pleurodonta [10].

The uncalibrated analyses here placed *Magnuviator ovimonsensis* as sister to (Crotaphytidae, Corytophanidae) ([9] scaffold) or to crown pleurodontans excluding Phrynosomatidae, Iguanidae and Anolidae ([15] scaffold). The calibrated analyses placed *Magnuviator* in a clade with Late Cretaceous taxa previously placed in Isodontosauridae, and those taxa were collectively the sister clade of crown-Pleurodonta. As with *Polrussia*, all sister taxon relationships of *Magnuviator* were poorly supported (less than 0.5 pp). None of the present analyses found a sister relationship between temujiniids and *Magnuviator ovimonsensis*, although that is not surprising given that Temujiniidae was placed here as the sister of all iguanians (like all analyses using the matrix from Smith [13]) instead of as a stem pleurodontan clade, as in many analyses that used the matrix of Gauthier *et al*. [21]. Either way, the placement of *Magnuviator ovimonsensis* as a stem pleurodontan in several analyses here is similar to some of the results of DeMar *et al*. [10].

The placement of *Magnuviator ovimonsensis* as a stem pleurodontan, if correct, is broadly consistent with the hypothesis that the ancestor of crown-Pleurodonta dispersed to North America from central Asia [26,92]. That said, based on both the present results and those of DeMar *et al*. [10], *Magnuviator* may be part of a central Asian clade of stem pleurodontans instead of being closer to the crown. Previous hypotheses generally entail the dispersal of the ancestor of crown-Pleurodonta, not multiple stem pleurodontans or crown pleurodontans, to the Western Hemisphere via the Bering land bridge. If *Polrussia mongoliensis* IGM 3/73 and *Magnuviator* are both correctly placed in the present analyses, then there may have been several pulses of dispersal or a single simultaneous dispersal across Beringia into North America of stem and crown pleurodontans. Based on fossil flora, potentially dinosaurs, and some palaeotectonic reconstructions, Beringia was a viable land migration corridor during the Late Cretaceous near the age of *Magnuviator* (approx. 75 Ma) [93–97].

#### 5.4.3. *Afairiguana avius*, the origin of anoles and considerations for node calibrations

*Afairiguana avius* was described by Conrad *et al*. [89] from the early Eocene Green River Formation of Wyoming, and placed via phylogenetic analysis in crown-Polychrotidae *sensu* Frost & Etheridge [19]. Most molecular phylogenies (e.g. [9,15,26]) indicate that the morphological hypothesis of Polychrotidae is polyphyletic, and the clades Polychrotidae (restricted to *Polychrus*), Anolidae and Leiosauridae are recognized instead, or the subfamily nomenclature of those clades if ‘Iguanidae' is preferred over ‘Pleurodonta' (see [20,24]). Though none of the three families form a grade or clade in phylogenomic trees, Leiosauridae and Polychrotidae are more closely related to each other than either are to Anolidae [9,15]. Within ‘Polychrotidae', *Afairiguana avius* was initially inferred to be in an unresolved trichotomy with Leiosaurinae and Anisolepinae [89]. In a combined-evidence analysis using an expanded morphological matrix, *Afairiguana* was placed in a polytomy with Anolidae and *Polychrus* [45]. In a more recent combined-evidence divergence-time analysis of UCEs and the matrix from Conrad [45], *Afairiguana* was placed as sister to Leiosauridae [8], and in a new morphological analysis, *Afairiguana* was placed as the sister of *Anolis* [98]. The reported diagnostic characters of *Afairiguana* include rugosities on the jugal, the presence of a discrete postfrontal, a posteriorly elongated dentary, proximally expanded and notched/fenestrated clavicles, postxiphisternal inscriptional ribs with midline contact, and caudal autotomy fracture planes anterior to the transverse processes [89]. The presence of the latter character state, often termed *Anolis*-type fracture planes, was first recognized by Smith [99] as offering a clue to the relationships of *Afairiguana*. In all of the analyses here, *Afairiguana avius* was inferred to be a stem anolid.

Previous studies indicated intraspecific variation in anole fracture-plane morphology [19,20,100], but fracture planes anterior to the transverse processes were recognized as being restricted to anoles. *Anisolepis grilli* and *Polychrus femoralis* were scored as having the *Anolis*-type fracture planes by Conrad *et al*. [89]. I was not able to examine either of those species and a comprehensive survey of fracture plane morphology in Pleurodonta was beyond the scope of this study. The specimen of *Anisolepis undulatus* and specimens of *Polychrus* (*acutirostris*, *gutturosus*, *marmoratus*) that I scored lack fracture planes altogether and so were scored as ‘-' for the character that addresses this morphology (116). The only specimens that I observed with *Anolis*-type fracture planes were extant anoles and *Afairiguana avius*. Based on the data I collected and data from previous studies that explicitly surveyed fracture plane morphology [19,20,100], *Anolis*-type fracture planes are autapomorphic of total clade Anolidae.

Based on this study and that of Bolet *et al*. [98], *Afairiguana avius* should be considered a member of total clade Anolidae instead of Leiosauridae. In the uncalibrated constrained analyses of this study, *Afairiguana* was nested in crown-Anolidae (the unusual *Anolis barbouri* was sister to the rest of Anolidae), and in the calibrated constrained analyses *Afairiguana avius* was sister to crown-Anolidae. Though posterior probability support placing *Afairiguana* within or as sister to Anolidae was low to moderate across analyses (either less than 0.5 or 0.79 pp), that inference was consistent across analyses.

There are no apomorphies that would clearly place *Afairiguana* within crown-Anolidae. Based on published divergence time analyses, the fossil, which was deposited *ca* 52 Ma, is slightly older [27] or slightly younger [101] than the age of crown-Anolidae. Although anoles are not found in Wyoming in the present day, the placement of *Afairiguana* in total clade Anolidae rather than Leiosauridae is more consistent with the modern biogeography of Pleurodonta and records of other fossil pleurodontans. Extant species of *Anolis* occur throughout Central America, the Caribbean, and much of North America and South America [101], whereas extant leiosaurids are restricted to southern South America, the Atlantic coastal forests of Brazil, and some areas in the eastern Amazon Rainforest [102,103]. No other fossil leiosaurids were described from North America (and see *Kopidosaurus* section below), but there are several other described putative stem anolids (*Anolbanolis*, *Paranolis*; [31,99]), some of which were included in the present analyses.

The presence of another anolid in the Eocene of Wyoming solidifies the hypothesis of Smith [31] that total clade Anolidae possessed a more northern distribution during the early Palaeogene compared with the distribution of crown anolids during the rest of the Cenozoic. The question remains, however, of whether these occurrences represent range expansions from the tropics and subtropics during the Palaeocene and Eocene as a result of climate tracking [31], or whether total clade Anolidae had a more northern and/or broader distribution ancestrally, and restriction to the tropics and subtropics occurred secondarily during the late Eocene or Oligocene. Additionally, some authors suggested that crown-Anolidae originated in South America [101]. Stem anoles in the Eocene of Wyoming do not contradict a potential origin of crown-Anolidae in South America. The phylogenetic position of Anolidae in Pleurodonta has varied substantially in recent phylogenomic trees, however, from relatively early-diverging (e.g. [15]) to more nested [9]. Clarity on the relationships of Anolidae will help to inform the biogeography of the crown and total clades.

Several divergence time studies incorporated *Afairiguana avius* as a node calibration or in FBD analyses (e.g. [8,15,104,105]). For the node calibrated analyses, the fossil was used to calibrate crown-Pleurodonta [104], the divergence between Anolidae and Leiosauridae [15], total clade Leiosauridae [105] or crown-Leiosauridae [8]. Fortunately, Welt and Raxworthy [8] performed analyses without the calibration that did not produce substantively different results from the analyses that included the calibration. For other analyses that treated *Afairiguana* as a leiosaurid (e.g. [105]), the resultant divergence times are unlikely to have been deleteriously affected by using *Afairiguana* as a calibration because the analyses did not produce outlier node ages with respect to studies that did not use *Afairiguana* as a calibration (e.g. [27]). For the analyses from Burbrink *et al*. [15], the use of *Afairiguana avius* as a calibration minimum was appropriate given that the taxon is a member of total clade Anolidae.

*Afairiguana avius* presents a different situation than many other fossil lizards that were erroneously used to anchor the minimum age of a node calibration. For most fossil lizards, inappropriate node calibrations result from the attribution of a fossil to a clade without performing or invoking an explicit phylogenetic analysis or apomorphic diagnosis to justify that placement [2,12]. For *Afairiguana*, attribution to Leiosauridae was the result of many phylogenetic analyses, including combined-evidence analyses with phylogenomic datasets. Therefore, these past issues with calibrations using *Afairiguana* resulted from not reassessing the underlying character data in the morphological matrix used to place the fossil, and similarly, not re-evaluating the describing paper and the characters used to make the qualitative diagnosis. Phylogenetic analyses are a cornerstone of palaeontology, but there is no substitute for examination of specimens and comparative material, whether via computed tomography, physical specimens, or even illustrations and photographs (as was done here for *Afairiguana*).

#### 5.4.4. *Babibasiliscus alxi* and the antiquity of crown-Corytophanidae

*Babibasiliscus alxi* was described by Conrad [45] from a well-preserved articulated skull from the early Eocene of Wyoming (*ca* 48 Ma). The new taxon was placed via phylogenetic analysis as sister to *Laemanctus* (casque-headed iguana), an extant corytophanid that presently occurs in forests of Mexico and upper central America [106]. Several divergence-time analyses estimated an early to middle Oligocene age (30–26 Ma) for crown-Corytophanidae and a latest Oligocene (24 Ma) divergence time between *Laemanctus* and *Corytophanes* (helmeted basilisk) [15,27]. Divergence-time analyses that used *Babibasiliscus alxi* as a node calibration for crown-Corytophanidae or in FBD tip-dating produced ages for crown-Corytophanidae of approximately 50 or 60 Ma, respectively [8].

Here, *Babibasiliscus alxi* was placed as a stem corytophanid in the uncalibrated analyses, as a stem crotaphytid (stemward from *Aciprion formosum*) in the calibrated analysis using the scaffold from Streicher *et al.* [9], and as sister to the clade (Leiocephalidae, Corytophanidae) in the calibrated analysis using the scaffold from Burbrink *et al*. [15]. All potential relationships were poorly supported in the analyses here (less than 0.5 pp). Previous divergence time analyses that did not include *Babibasiliscus alxi* as a calibration and the results of the present study suggest that placement in crown-Corytophanidae is incorrect. Attribution of *Babibasiliscus alxi* to total clade Crotaphytidae is conceivable and the presence of a stem crotaphytid in Wyoming during the early Eocene would not be surprising given the modern distribution of Crotaphytidae in North America (figure 1) and the present description of AMNH FR 11400. *Babibasiliscus alxi* has at least two morphological features—a posteroventral process of the jugal and a jugal that is exposed above the orbital process of the maxilla—that are more consistent with Crotaphytidae than Corytophanidae (see [13]). On the other hand, the fossil has a prefrontal-lacrimal groove, which was considered an autapomorphy of Corytophanidae [13,45], and lacks palatine teeth, the presence of which is an apomorphy of Crotaphytidae [13,38]. I suggest that *Babibasiliscus alxi* is a stem corytophanid or a stem crotaphytid, but there is uncertainty based on the present analyses.

#### 5.4.5. *Kopidosaurus perplexus*: potentially a corytophanid?

*Kopidosaurus perplexus* was described from a mostly complete and partially articulated skull collected from the early Eocene Willwood Formation of the Bighorn Basin, Wyoming [69]. The fossil, YPM 8287, presented an interesting combination of features, including an open Meckelian groove and a squamosal that lacks a dorsal (ascending) process. A suite of phylogenetic analyses using two matrices and several phylogenomic constraints consistently placed *Kopidosaurus* within crown-Pleurodonta, but produced divergent results with equivocal Bayes factor support regarding the relationships of the new taxon with respect to the family-level crown clades [69]. Based on the results of the phylogenetic analyses, Scarpetta [69] suggested that *Kopidosaurus* might be related to Crotaphytidae and Corytophanidae *or* to Opluridae, Leiosauridae and Hoplocercidae.

One other interesting feature of YPM 8287, a parietal table that exhibits a ‘y' or a ‘v' shape, was previously coded as ‘?' because the presence of a posterior crest of the table could not be determined and so a single character state could not be assigned [69]. The former configuration is present in Corytophanidae, Anolidae and Iguanidae, and the latter in Hoplocercidae and Iguanidae (this paper; [13]). For the present study, I recoded the character that addresses that morphology as polymorphic, rather than unknown, to attempt to better elucidate the relationships of *Kopidosaurus*. *Kopidosaurus* was here inferred to be a stem corytophanid in all analyses with topological constraints, but was placed as a stem hoplocercid in the unconstrained uncalibrated analysis. The results here are less chaotic than those of Scarpetta [69] and were not well-supported (less than 0.5 pp), but recapitulate the idea that *Kopidosaurus* exhibits derived morphologies that are consistent with Corytophanidae and Crotaphytidae or Hoplocercidae. However, I suggest here given the new topologically constrained analyses that included the revised character scorings that attribution to Hoplocercidae or the least inclusive clade that includes Hoplocercidae, Opluridae and Leiosauridae would be in error, and that any shared characteristics between that clade and *Kopidosaurus* result from homoplasy. Placement of *Kopidosaurus perplexus* in total clade Corytophanidae is reasonable but should still be considered ambiguous.

Several other putative stem hoplocercids were described from the Cretaceous or Palaeogene of North America. *Cypressaurus* from the Eocene and Oligocene of Saskatchewan and North Dakota [42] and *Pariguana lancensis* from the Late Cretaceous of Wyoming were reported to be putative stem hoplocercids [32,36]. Subsequent phylogenetic analyses [10] indicated that *Pariguana* is probably not a hoplocercid, although the one known specimen is a partial mandible, so evidence for attribution to any extant family may be deficient. On the other hand, Smith [32] noted some similarities between *Cypressaurus* and Hoplocercidae but was cautious about suggesting a close relationship between the two taxa. The systematic position of both taxa should be considered uncertain. I would argue that the anteroposterior narrowness of the facial process of the maxilla that is present in *Cypressaurus*, which was interpreted by Smith [32] as a potentially shared derived feature of that taxon with Hoplocercidae, could just as easily indicate an exclusive relationship with Crotaphytidae. Extant species of *Crotaphytus* can also have a narrow facial process (figure 5), although perhaps not as narrow as that of extant hoplocercids. Both *Cypressaurus* and *Pariguana lancensis* require further study and neither should be considered part of Hoplocercidae.

### 5.5. Clarifications on the morphology of the infraorbital foramen in Late Cretaceous iguanians

The morphology of the infraorbital foramen is a potentially useful character for identifying and systematically placing fossil iguanians. Some iguanians (e.g. some members of Iguanidae) retain the probable ancestral character state in which the infraorbital foramen is bounded entirely by the lateral processes of the palatine. In some pleurodontans, the foramen is incompletely bounded by the lateral processes (e.g. Crotaphytidae), and for others the posterolateral process of the palatine is nearly or completely absent and so the maxilla has a greater contribution to the boundary of the foramen (e.g. Phrynosomatidae). Members of Isodontosauridae and Temujiniidae were previously interpreted as having an infraorbital foramen entirely in the palatine [21,31], and the state was a hypothesized apomorphy of Isodontosauridae [21]. Several of those taxa were CT-scanned for the Squamate Tree of Life project ([21]; e.g. *Isodontosaurus*, *Temujinia*, *Zapsosaurus*, *Polrussia*). None of those scans were segmented and all character scorings were taken from volume renderings. I found that the morphology of the infraorbital foramen was difficult to visualize in the CT volume renderings for each of those taxa. The structure was obscured by other bones or by matrix that could not be removed via grey-scale value manipulation without removing part of the relevant morphology. I partially segmented the palatines of the scans of *Isodontosaurus*, *Temujinia*, *Polrussia* (the palatines are degraded in the specimen of *Zapsosaurus*) and determined that only *Isodontosaurus* retains the ancestral infraorbital foramen morphology. In *Temujinia*, the infraorbital foramen is bounded by lateral and posterolateral processes of the palatine, while in *Polrussia* the posterolateral process is absent.

### 5.6. The Cretaceous and Palaeogene record of fossil pleurodontans in North America

Crown pleurodontans from the Late Cretaceous of North America are unknown [10] or rare [36]. Based on my osteological observations and phylogenetic analyses, I suggest that the use of more targeted matrices, combined-evidence or topologically constrained analyses, and the estimation of a more robust phylogenetic hypothesis for Pleurodonta, may illuminate additional crown pleurodontans from the Late Cretaceous and early Cenozoic.

Fossil lizards are poorly described from the Oligocene compared with the Eocene and the Neogene, although glyptosaurine anguids are still relatively abundant though not diverse (see [107,108]). Few fossil pleurodontans are published from the Oligocene of North or South America [40], although many have been collected (SG Scarpetta 2023, personal observation). On the Paleobiology Database (PBDB)—which is by no means a comprehensive database of published fossil lizard occurrences—there are only 10 listed occurrences of pleurodontans in North America, two in South America and four in Europe from the Oligocene [109]. Extinct pleurodontans include enigmatic taxa like *Cypressaurus* and ‘*Crotaphytus' oligocenicus* (see [40]), which currently offer little in the way of biogeographic or ecological information given their uncertain systematic position. Future research efforts should target the Oligocene (and the Palaeocene, which is also poorly known) for descriptive efforts of fossil pleurodontans.

## 6. Conclusion

When identifying and systematically placing fossils, we cannot fully know all relevant morphological features, true evolutionary relationships, or the best method for evaluating the relationships of extinct taxa given the available data. It follows that fossil identifications will never be immutable, but they can be stable. Based on explicit matrix selection and careful revision, relatively broad taxon sampling, and use of appropriate methodology and topology, the placement of *Aciprion formosum* AMNH FR 11400 in total clade Crotaphytidae should be resilient to any subsequent hypotheses of iguanian lizard phylogeny. I emphasize that stability is contingent on appropriate matrix choice given previous uncertainty of the relationships of AMNH FR 11400 using other matrices. More broadly, the results of this study will be re-evaluated in the future in combined-evidence analyses that use phylogenomic datasets, which will hopefully better approximate the evolutionary relationships of Iguania and thus better and/or more confidently place the extinct taxa.

## Data Availability

The data are provided in electronic supplementary material [139].

## Appendix A. Character revisions, clarification and comments

I added new characters numerically to the end of the matrix of Smith [13] such that the matrix is not renumbered, but still provide a numerical designation based on bone that is consistent with the rest of the matrix (i.e. ‘Dentary VIII' for new character 155). I also retained all formatting conventions from Smith [13].

**A.1. Character 33. Postfrontal II. Comment on scores for iguanid lizards**

No crown pleurodontans were considered by Smith [13] to have a postfrontal with a sufficiently well-developed posterior process to code state 1 for character 33, i.e. the clasping morphology of the postfrontal found in many non-iguanians lizards and in the hypothesized iguanian clade Temujiniidae. I have observed anguids (e.g. *Gerrhonotus parvus*) with a posterior process of the postfrontal that is comparable in relative size to those of a few iguanids (*Conolophus*, *Ctenosaura*, *Sauromalus*), and so have coded those iguanids as state 1.

**A.2. Character 41. Squamosal I. Comment on scores for pleurodontan lizards**

The presence of a dorsal (ascending) process of the squamosal was previously considered to be an apomorphy of Iguania [13,21]. I have also only observed that morphology in iguanians, but I have examined several crown pleurodontans that lack a dorsal process (e.g. *Dipsosaurus dorsalis* YPM Herr 14376; *Petrosaurus mearnsi* TxVP M-9609; *Uma paraphygas* TNHC 30596).

**A.3. Character 34 and 35. Postfrontal III and IV. Scoring changes**

Several taxa (e.g. crotaphytids, agamids) previously scored ‘?' (absence of postfrontal) for character 32 (presence, absence and fusion of postfrontal) had non-‘?’ character scores for character 34 and 35. The states for characters 34 and 35 are contingent on the presence of a separate postfrontal or apparent fusion of the postfrontal to the postorbital or frontal. Those taxa now have character scores of ‘?' for characters 34 and 35.

**A.4. Character 93. Dentition VI. Character revised**

Simões *et al.* [68], no. 210. Position of posterior dentary teeth relative to the apex of the labial wall of the dentary (0) lingual, (1) apical, (2) apicolingual.

Recently, Simões *et al*. [68] reframed an often-used dental character in squamate phylogenetics with states generally termed ‘pleurodonty', ‘acrodonty' and ‘pleuroacrodonty’. Their new lepidosauromorph matrix invoked two separate transformations: the position of the teeth relative to the apex of the labial wall of the dentary, and whether the teeth are ankylosed to the dentary/maxilla. For phylogenetic assessments of squamates with ‘pleurodont' teeth the distinction is less important, although it should be implemented regardless. For acrodontan iguanians, however, the distinction is important. The position of the posterior dentary teeth with respect to the dentary labial wall differs between agamids (apicolingual) and chameleons (apical), whereas ankylosis is present in all acrodontans, differentiating that group from pleurodontans and most other squamates. The position of the teeth varies in extinct acrodontans as well. I follow Simões *et al*. [68] in splitting the original character into two characters. I also add their character describing the position of the anterior dentary teeth, which also varies among extant and extinct acrodontans.

**A.5. Character 153. Dentition VII. New**

Simões *et al.* [68], no. 211. Ankylosis of posterior dentary teeth to the apex of the labial wall of the dentary (0) absent, (1) present.

**A.6. Character 154. Dentition VIII. New**

Simões *et al.* [68], no. 216. Position of anterior dentary teeth relative to the apex of the labial wall of the dentary (0) lingual, (1) apical, (2) apicolingual.

**A.7. Character 94. Dentary I. Character state added**

Etheridge [110], Etheridge and de Queiroz [18] no. 11–12, Lang [106] no. 31, Frost and Etheridge [19] no. 20, Gauthier *et al*. [21] no. 372. Meckelian sulcus (0) open and unrestricted by the ventral border of the dentary (= inframeckelian lip), (1) open but restricted by dorsal curl of the ventral margin of the dentary (inframeckelian lip) (new state), (2) dorsal and ventral margins of dentary in contact but not fused, enclosing a canal, or (3) dorsal and ventral margins indistinguishably fused, enclosing a canal.

**A.8. Remarks**

The wording of the revised character is intended to be close to Smith [13]. The character formulation now reflects the character states used for the equivalent character (372) in Gauthier *et al*. [21]. The new state was added to accommodate the difference between an open and unrestricted Meckelian groove and an open but restricted Meckelian groove, in which the ventral margin of the dentary (= inframeckelian lip *sensu* [58]) curls dorsally to constrain the Meckelian groove without contacting the upper margin of the dentary (= suprameckelian lip). The former state is probably ancestral [21] and is present in many squamates, including all acrodontans and Hoplocercidae among iguanians, and the latter state is found in several pleurodontans (e.g. some phrynosomatids, crotaphytids, corytophanids and liolaemids) and in some sphenomorphine skinks, but to my knowledge does not occur in other squamates. Anterior restriction of the Meckelian groove by dorsoventral expansion of the suprameckelian lip occurs in some teiids [111].

**A.9. Character 95. Dentary II. Character state ‘0' removed**

All taxa scored ‘0' or ‘1' in character 94 (i.e. character state 0 in the original scoring scheme in [13]) are now scored ‘?'.

This character differs from the previous character in addressing the degree of closure of the Meckelian groove rather than the presence of closure. A character state was included for character 95 in the original matrix for an open Meckelian groove (state ‘0'), but the inclusion of that state added additional weight to the open groove morphology, which was already addressed in character 94. For that reason, specimens possessing an open Meckelian groove should not be scored for this character and are now scored as ‘?'. The character states '1' and ‘2' are left as is both in the description and scoring.

**A.10. Character 97. Dentary IV. Scoring clarification**

For some examined agamids (e.g. *Sitana*, *Calotes*), the anteromedial process of the coronoid and the splenial articulate entirely medial to the suprameckelian lip (state 0), but the process itself does not extend past the first or second distalmost tooth position, which is a qualifier used by Smith [13] for the medial and lateral articulation states. These specimens are scored as state ‘0', because regardless of the number of tooth positions spanned by the articulation, the articulation occurs entirely medially.

**A.11. Character 99. Dentary VII. Character revised and split**

Previously based on Etheridge and de Queiroz [18], Frost *et al*. [20] no. 63. New character framing partially based on Gauthier *et al*. [21] no. 364. Posterior extent of the surangular (dorsal) process of the dentary; (0) surangular process of the dentary posterior extent is anterior to or at the apex of the coronoid process of the coronoid, (1) surangular process of the dentary posterior extent is posterior to the apex of the coronoid process of the coronoid but does not approach the anterior extent of the quadrate articulation surface of the articular, (2) = surangular process of the dentary is level with or past the articular surface for the quadrate.

Phylogenetic matrices for iguanian lizards previously assessed the relative length of the dentary with respect to the mandible regardless of which structure of the dentary extended farthest posteriorly [13,18,20]. The relative length of the dentary incorporates two separate morphological features, the posterior extent of the surangular process and the posterior extent of the angular process. The relative lengths of those processes with respect to the mandible and the coronoid apex are decoupled among many iguanians, and so should be considered separate transformations. Thus, character 99 is reframed and a new dentary character (here labelled character 155) is added. For example, in many *Phrynosoma* (e.g. *Phrynosoma asio*) and in some iguanids (e.g. *Sauromalus ater*), the angular process but not the surangular process extends posterior to the coronoid apex, whereas in crotaphytids and most corytophanids the reverse is true. In other iguanians such as Anolidae, Opluridae, Liolaemidae and most members of Acrodonta, both processes exceed the coronoid apex but do not approach the articular surface for the quadrate. In several acrodontans and *Sphenodon*, the angular process but not the surangular process reaches the level of quadrate articular surface. For several Cretaceous iguanians (e.g. *Polrussia*, *Zapsosaurus*) neither process reaches the coronoid apex. In iguanians, the surangular process is the larger of the two posterodorsal processes of the dentary, and the coronoid process (the dorsalmost process) is relatively less developed in its posterior extent and overall size.

In some anguids (e.g. anguines, some *Abronia*), the coronoid process is well-developed and extends relatively far posteriorly, and in several gerrhonotines, such as most *Elgaria*, the surangular process is absent [112]. Regardless, no posterior process of the dentary exceeds the coronoid apex in anguids.

**A.12. Character 155. Dentary VIII. New**

Based on Gauthier *et al*. [21] no. 369. Posterior extent of the angular (ventral) process of the dentary; (0) angular process of the dentary posterior extent is anterior to or at the apex of the coronoid process of the coronoid, (1) angular process of the dentary posterior extent is posterior to the apex of the coronoid process of the coronoid but does not approach the anterior extent of the quadrate articulation surface of the articular, (2) = angular process of the dentary is level with or past the articular surface for the quadrate.

## Appendix B. Temporal constraint and locality information for extinct iguanians included as terminal taxa

**B.1. *Aciprion formosum***

See main text.

**B.2. *Afairiguana avius***

*Afairiguana avius* FMNH PR 2379 was collected from the Warfield Springs locality at Fossil Lake in southwestern Wyoming [89]. Fossil Lake is part of the Fossil Butte Member of the Green River Formation [89]. ^40^Ar/^39^Ar dating from an ash tuff near the top of the Fossil Butte Member yielded an age of 51.97 ± 0.09 Ma [113]. The Fossil Butte Member was deposited entirely with Wa7 (the seventh stage of the Wasatchian NALMA; [114]), which extends as early as 52.8 Ma based on ^40^Ar/^39^Ar dates from sanidine in the Upper Willwood Formation [115,116]. The age range of *Afairiguana avius* FMNH PR 2379 is 51.88–52.8 Ma.

**B.3. *Anchaurosaurus gilmorei***

Specimens of *Anchaurosaurus gilmorei* (IVPP V10028 was scored here) were collected from the Djadokhta Formation in the Gobi Desert of Mongolia [92]. The Djadokhta Formation was suggested to extend 71–75 Ma during the latest Campanian based on a tentative correlation between magnetozones at the Flaming Cliffs locality and the end of chron 33 and (presumably the beginning) of chron 32 [117]. The Campanian was previously considered to extend to around 71 Ma [118] but presently is considered to extend to 72.1 Ma [119]. The age range of the probably overlying and younger Barun Goyot Formation is more broadly defined as Campanian, 72.1–83.6 Ma [86,119,120]. However, there is no definitive contact between the Djadokhta and Barun Goyot formations [120] and the formations may actually interfinger in some places [121]. I conservatively assign a Campanian age (72.1–83.6 Ma) to all of the Gobi Desert fossils collected from the Djadokhta and Barun Goyot formations.

**B.4. *Anolbanolis banalis***

Specimens of *Anolbanolis banalis* (holotype UCMP 400150 and other specimens from the describing paper were scored) were collected from locality UCMP V99019 of the lower Willwood Formation in the Bighorn Basin of northcentral Wyoming [31]. That locality occurs within the carbon isotope excursion at the Palaeocene–Eocene boundary. The base of the excursion is at 56 Ma and the event lasted around 170 kyr [52,53,122]. The age range of *Anolbanolis banalis* is 55.83–56 Ma.

**B.5. *Anolbanolis geminus***

Specimens of *Anolbanolis geminus* (holotype USNM 527980 and other specimens from the describing paper were scored) were collected from Dorsey Creek Quarry (USNM locality D2035Q) in the lower Eocene Willwood Formation in the Bighorn Basin, Wyoming [99]. The Dorsey Creek Section of which the locality is a part is in chrons C24r-C24n.3n [123] and is within Wa5 [99,124,125], whose lower boundary is within approximately 250 kyr of the C24r-C24n.3n boundary ([123]). Thus, the minimum age of the fossils is 53.42 Ma (beginning of chron C24n.3n; [54]) and the maximum age is about 53.67 Ma [123].

**B.6. *Armandisaurus explorator***

*Armandisaurus explorator* AMNH FR 8800 was collected from the Skull Ridge Member of the Tesuque Formation in Santa Fe County New Mexico [126]. ^40^Ar/^39^Ar dating of a tuff near the top of the member yielded an age of 15.3 ± 0.05 Ma, and the same method produced an age of 15.5 ± 0.07 Ma from a tuff near the bottom of the member [127]. The age range for *Armandisaurus explorator* AMNH FR 8800 is 15.25–15.57 Ma.

**B.7. *Babibasiliscus alxi***

*Babibasiliscus alxi* UWBM 89090 was recovered from Lucky Lizard Locality (UWBM C1046) in Uinta County of southwestern Wyoming [45]. UWBM C1046 is in the Blacks Fork member of the Bridger Formation (also termed Bridger B) in the Green River Basin [45]. The Blacks Fork member was deposited during the Br2 biochron of the Bridgerian NALMA, which spans chrons C22r–C21n, 47.91–49.04 Ma [116,128].

**B.8. *Calumma benovskyi***

*Calumma benovskyi* KNM-RU 18340 was collected from the Hiwegi Formation in the Kisingiri sequence of western Kenya. K-Ar ages near the top and bottom indicate ages of 16.9 ± 0.2 and 21 ± 0.3 Ma, respectively [129]. The age range of *Calumma benovskyi* is 16.7–21.3 Ma.

**B.9. *Ctenomastax parva***

*Ctenomastax parva* (the specimen scored here is IGM 3/62) has been collected from the Late Cretaceous Djadokhta and Barun Goyot Formations [86] and so is assigned an age range of 72.1–83.6 Ma (see *Anchaurosaurus gilmorei* section above).

**B.10. *Gambelia corona***

*Gambelia corona* LACM 42880 was recovered from the Palm Spring Group at locality LACM 7058, which is in the Anza Borrego Desert of southern California. The group was deposited during the Pliocene and Pleistocene and the locality is in strata correlated with chron C2Ar ([130]; L Murray 2021, personal communication). Chron C2Ar and thus the age of the fossil spans 3.596–4.187 Ma [131].

**B.11. *Geiseltaliellus maarius***

*Geiseltaliellus maarius* (holotype HLMD-Me 10207 and other specimens from Smith [13]) and other specimens of this species were collected from the Lagerstätte of the Messel Formation near Frankfurt, Germany. The Lagerstätte event occurred 47.8 ± 0.2 Ma, but extrapolation based on height above the base of the formation and sedimentation indicates that the fossiliferous layers from which fossil *Geiseltaliellus* were recovered were deposited at about 47 Ma [132]. I conservatively assign an age range of 47–48 Ma. Recently, an earlier record of *Geiseltaliellus* was published from the earliest Eocene of Belgium [133], but because no species assignment was made for those specimens, I retain the later age for *Geiseltaliellus maarius*.

**B.12. *Isodontosaurus gracilis***

This enigmatic Cretaceous iguanian (the specimen IGM 3/84 was scored here) has been collected from the Djadokhta Formation [86]. I assign an age range of 72.1–83.6 Ma (see *Anchaurosaurus gilmorei* section above).

**B.13. *Kopidosaurus perplexus***

The minimum age of *Kopidosaurus perplexus* YPM 8287 is 52.47 Ma (see [69] for a discussion). The locality of YPM 8287 (YPM 24) was deposited entirely within Wa7 [115,116], so the maximum age of the fossil is 52.8 Ma.

**B.14. *Magnuviator ovimonsensis***

Specimens of *Magnuviator ovimonsensis* (holotype MOR 6627, referred specimen MOR 7042 were both scored) were collected from the Egg Mountain Locality in the Two Medicine Formation in northwestern Montana [10]. This Late Cretaceous locality has been dated to 75.5 ± 0.4 Ma based on ^40^Ar/^39^Ar analysis [134], so the age range of the species is 75.1–75.9 Ma.

**B.15. *Mimeosaurus crassus***

Like *Isodontosaurus*, *Mimeosaurus crassus* (IGM 3/74 and 3/76 were scored) has been collected from the Djadokhta Formation [86] and so is assigned an age range of 72.1–83.6 Ma (see *Anchaurosaurus gilmorei* section above).

**B.16. *Oreithyia oaklandi***

*Oreithyia oaklandi* (holotype PTRM 5198 and other specimens from describing paper were scored) is known from the Medicine Pole Hills fauna of the Chadron Formation in southwestern North Dakota [32]. An ash from the Chadron Formation was dated via ^40^Ar/^39^Ar to 35.41 ± 0.14 Ma [135]. Extrapolation via sedimentation rate provided an age of 35.2 Ma for the fauna [32]; I use that value as the minimum age and 35.55 Ma as the maximum age for these fossils.

**B.17. *Phrynosomimus asper***

*Phrynosomimus asper* (IGM M81 was scored here) has been collected from the Late Cretaceous Djadokhta and Barun Goyot Formations [86], and so is assigned an age of 72.1–83.6 Ma (see *Anchaurosaurus gilmorei* section above).

**B.18. *Polrussia mongoliensis***

*Polrussia mongoliensis* IGM 3/73 was collected from the Late Cretaceous Barun Goyot Formation from the Khulsan locality [86]. The fossil is assigned an age of 72.1–83.6 Ma (see *Anchaurosaurus gilmorei* section above).

**B.19. *Priscagama gobiensis***

Specimens of *Priscagama gobiensis* (holotype ZPAL MgR/III-32) were collected from the Barun Goyot Formation [86], and so the taxon is assigned an age of 72.1–83.6 Ma (see *Anchaurosaurus gilmorei* section above).

**B.20. *Pumilia novaceki***

Specimens of *Pumilia novaceki* (holotype LACM 13739 and LACM 78310 were scored here) were collected from the Palm Springs group at several localities (LACM 65116, 6661) spanning chron C2Ar to chron C2An.2r ([130]; L Murray 2021, personal communication), 3.22–4.187 Ma [131].

**B.21. *Saichangurvel davidsoni***

The type and only known specimen of *Saichangurvel davidsoni*, IGM 3/858, was collected from the Djadokhta Formation [136] and so is assigned an age of 72.1–83.6 Ma (see *Anchaurosaurus gilmorei* section above).

**B.22. *Sauropithecoides charisticus***

Fossils of *Sauropithecoides charisticus* (holotype PTRM 1841 and other specimens from the describing paper were scored) were collected from the Medicine Pole Hills fauna of the Chadron Formation in southwestern North Dakota [32] and so are assigned an age range of 35.2–35.55 Ma (see *Oreithyia oaklandi* section).

**B.23. *Suzanniwana patriciana***

Fossils of *Suzanniwana patriciana* (holotype UCMP167664 and other specimens from describing paper were scored here) were collected from locality UCMP V99019 of the lower Willwood Formation, and so are given an age range of 55.83–56 Ma (see *Anolbanolis banalis* section above).

**B.24. *Suzanniwana revenata***

*Suzanniwana revenata* (holotype UCMP 167682 and other specimens from describing paper) is known from several localities in the early Eocene Wasatch Formation in Wyoming. A biochronological correlation established that the fossils are probably within the Wa5 and Wa6 biochrons of the Wasatchian NALMA [137], and so this taxon is assigned an age range of 52.76–53.35 Ma. That range is subject to change in future analyses should those biochrons be modified or new data provide a different age estimate.

**B.25. *Temujinia ellisoni***

Known specimens of *Temujinia ellisoni* (IGM 3/63 was scored here) were collected from the Djadokhta Formation [86] and so are given an age range of 72.1–83.6 Ma (see *Anchaurosaurus gilmorei* section above).

**B.26. *Queironius praelapsus***

Fossils of *Queironius praelapsus* (holotype PTRM 19499 and other specimens from the describing paper were scored) are known from the Medicine Pole Hills fauna of the Chadron Formation in southwestern North Dakota [32] and so are assigned an age range of 35.2–35.55 Ma (see *Oreithyia oaklandi* section).

**B.27. *Zapsosaurus sceliphros***

Specimens of *Zapsosaurus sceliphros* (IGM 3/71 was scored here) were collected from the Djadokhta Formation [86] and so are assigned an age of 72.1–83.6 Ma (see *Anchaurosaurus gilmorei* section above).

**B.28. Root**

The tree root age was established using a combination of fossil data and results of previous divergence time analyses. The outgroups include several non-iguanian squamates, including a gecko. The oldest gecko is a stem taxon, *Eichstaettisaurus schroederi* [68], which was probably deposited during the Jurassic between 150 and 155 Ma based on ammonite biostratigraphy [138]. I used an offset exponential prior, for which 150 Ma was used as the offset, and used the median age of crown-Squamata from a recent phylogenomic divergence time analyses, 193.2 Ma [15], as the mean.
